# Target, silence, replace: a review on RNA-based drugs in modern medicine

**DOI:** 10.3389/fcell.2026.1879681

**Published:** 2026-09-01

**Authors:** Poonam Mundlia, Suraj Pratap Singh, Pritiman Pothal, Tanya Thakur, Reema Kathuria, Sudhanshu Maurya, Akanksha Sharma, Honey Goel, Ankur Pandey, Pavitra Ranawat, Gurpal Singh, Ravi Pratap Barnwal

**Affiliations:** 1 Department of Biophysics, Panjab University, Chandigarh, India; 2 University Institute of Pharmaceutical Sciences, Panjab University, Chandigarh, India; 3 Department of Biotechnology, Panjab University, Chandigarh, India; 4 Department of Pharmaceutics, University Institute of Pharmaceutical Sciences and Research, Baba Farid University of Health Sciences, Faridkot, India; 5 Department of Chemistry, Panjab University, Chandigarh, India

**Keywords:** antisense oligonucleotides, aptamer, messenger RNA, nanoparticle delivery, RNA interference, RNA therapeutics

## Abstract

RNA therapies have evolved into a revolutionary approach in contemporary medicine for treating various diseases by directly targeting RNA molecules engaged in disease pathogenesis. These therapeutic agents regulate biological processes through diverse mechanisms, including modulation of RNA function and gene expression. Medical applications of RNA are greatly enhanced by its structure, adaptability, and capacity for targeted binding. Among these traits is its ability to bind to certain molecules unique to those chemicals. RNA-based treatments have emerged from advancements in the production, modification, and cellular transport of RNA molecules. Several RNA drugs have been approved whereas some are under trial for few diseases. RNA therapeutics can function at the level of RNAs, DNAs and proteins. The evolution of mRNA vaccines during the COVID-19 epidemic emphasizes the exciting potential of RNA therapies in the treatment of diseases. This article provides a comprehensive overview of the several forms of RNA therapies, including small-interfering RNA (siRNA), messenger RNA (mRNA), and antisense-oligonucleotides (ASOs), together with information on their action mechanisms and delivery strategies that improve cellular absorption and shield RNA molecules from degradation. Further, CRISPR-based editing of the genome can be employed for modification of target RNA sequences for various disorders. Development of RNA aptamers have also been identified as pivotal RNA-therapeutic candidate. Additionally, we have explained mechanistic details and examples of drugs approved for RNA therapy. Emphasizing their potential to enhance patient outcomes and fulfil unmet medical requirements, we also highlight the clinical development of RNA therapies in treating cancer and other infectious diseases.

## 1 Introduction

Ribonucleic acid (RNA) has been the subject of extensive research for several decades owing to its fundamental roles in cellular processes and gene regulation. Subsequent studies have revealed that certain RNA molecules possess catalytic activity and perform diverse biological functions beyond their classical role in protein synthesis. The first studies primarily focused on understanding the similarities between RNA and deoxyribonucleic acid (DNA) in terms of structure and function. RNA quickly became important as a key part of translation, which is the second step in processing genetic information after transcription. This process is what makes proteins and codes for genes (Sanger et al., 1976; Mattick and Amaral, 2023). Over time, researchers began to explore the vast diversity of RNA and its complex interactions with biological systems. In the last 60 years, five Nobel Prizes have been given for RNA research. This shows how important the molecule is in biology and chemistry and how it can be used in many ways in medicine (Kim, 2023). RNA technology has been around for a long time, but it has undergone significant changes over the years. At first, it was mostly about basic research, with a focus on RNA’s role in cellular processes. Currently, RNA has practical applications in medicine, including the development of mRNA vaccines, RNA-based drugs, and technologies that silence genes. Due to their distinct physicochemical and physiological properties, RNA-driven therapies hold great promise compared to conventional protein-targeted and DNA-based therapies (Zhu et al., 2022; Kramps and Probst, 2013). Major discoveries such as RNA catalysis, splicing and silencing have led to exploration of various other functions of RNA as they are honoured with Nobel prize. This has caused propelling of RNA field to delve deeper into therapeutic areas leading to harnessing of RNA molecules for even undruggable targets in the body. Discovering Small molecules that specifically target individual proteins has been a longstanding approach used by drug developers. These small molecules basically act as antagonists by binding to certain proteins like enzymes or receptors and thus, show a pharmacological effect. Possibility of oral administration, better pharmacokinetics, simple synthesis and ability to penetrate cell membranes are some of its potential advantages (Peterson and Liu, 2023). Despite their advantages, small molecules also have notable shortcomings. While they are good in getting enzymes blocked, as receptor ligands or in changing allosteric sites, they are not so good when it comes to discriminating protein-protein interactions necessary for many biological processes. As not all small compounds are able to effectively inhibit disease associated proteins, this obstacle is a major problem (Zhong et al., 2024; Gurevich and Gurevich, 2014). Accordingly, the search for therapeutic options that do not utilize small molecules is now more and more pressing. A more efficient route of drug development might come from targeting the RNA that encodes these proteins, rather than the proteins themselves. Drugs based on advanced biological macromolecules, including the nucleic acid-based therapeutics and monoclonal antibodies (mAbs), are increasingly being used in the pharmaceutical market today (Yamada, 2021; Niazi, 2023).

Nucleic acid-based therapies, in particular, are gaining significant momentum as the focus shifts toward more targeted and potent treatments (Mollocana-Lara et al., 2021; Damase et al., 2021). Examples of these therapeutic agents include oligonucleotides (Egli and Manoharan, 2023), plasmid DNA (Ghaffarifar, 2018), mRNA (Żak and Zangi, 2025), ribozymes (Roshan Mehr et al., 2024), RNA aptamers, and RNAi-related nucleic acids such as small interfering (siRNA) and microRNA (miRNA) (Dykxhoorn and Lieberman, 2006; Hansen et al., 2013). DNA oligonucleotide Fomivirsen, which was authorised for therapeutic use, was the first nucleic acid molecule used for medical purposes (Tosar, 2024). Subsequently, in 2018, the U.S. Food and Drug Administration (FDA) granted approval for the first RNA-based medicinal oligonucleotide, Patisiran, marketed under the brand name Onpattro. RNA-based therapeutics pose many limitations of other therapies; they are easy to synthesize on a large scale with consistent quality, making them an affordable and highly adaptable treatment option applicable to any stage of the RNA life cycle (Makkar, 2025). Since 2016 only, the RNA market started blooming with RNA based products and technologies being approved. Spinraza, an antisense oligonucleotides (ASOs) medication employed for the treatment of spinal muscular atrophy (SMA) was approved in 2016 and it took nearly 6 years for it to have enormous sales. The surge in RNA-based therapy happened only after 2016 with the development and approval of new products. At its core, RNA therapeutics is based on modulating the process of protein function and/or expression by directly targeting proteins, their coding RNAs, or the instruction set for making those proteins (Cronin and Yu, 2025). Three primary methodologies are used (a) inhibition of the protein product using aptamers (b) targeting the native RNA by ASOs, siRNA or miRNA mimics and (c) generate a target protein upon mRNA delivery (Metkar et al., 2024). RNA’s distinct structural and biochemical characteristics make it a highly programmable molecule, largely because of the well-understood principles of complementary base pairing, which govern the specificity and affinity of RNA interactions. As opposed to conventional drug development, which entails a long and expensive discovery process to find lead compounds, for RNA medicines, leads can be rationally designed after target identification (Sparmann and Vogel, 2023). RNA therapy development can be assisted by the use of artificial intelligence that can aid the selection of favourable and promising candidates. RNA-based treatments have been created for a range of diseases from infections to genetic disorders and cancer (Jeong and Jeong, 2025). RNA therapies can efficiently be used for diseases that are incurable and the ones that cannot be treated with conventional drugs (Bajan and Hutvagner, 2020; Sridharan and Gogtay, 2016).

Despite biological challenges, numerous clinical trials have shown the promise of RNA-based treatments. However, large and negatively charged RNA molecules cross the lipid bilayer of cell membranes with great difficulty, which generally allow only small and hydrophobic molecules to diffuse passively. Moreover, the endogenous defense system of the body including RNases, exonucleases, endonucleases as well as innate immune response pose considerable challenges in expeditiously delivering exogenous RNA. Negating these immunogenic obstacles is crucial to the successful delivery of therapeutic RNA. Delivery of RNA therapeutics using nanoparticle assisted strategies has generated immense enthusiasm owing to their potential in addressing these challenges. These delivery systems protect RNA drugs from the immune system and transport them safely to cells (Wang, 2020). RNA therapeutics have the potential to speed up the delivery of treatment to patients, make medicine more personalized, and target hard-to-reach areas (Batisani, 2025; Coutinho et al., 2019). Bulk production, efficient delivery methods, and immunogenicity properties are certain initial challenges for RNA-based drugs that are now being considered (Damase et al., 2021). Several methods are being devised for achieving better target specificity, enhanced stability and increased circulation time (Kaczmarek et al., 2017; Zhou et al., 2026). Despite several advancements, the challenges with effective delivery and manufacturing still exist.

This paper covers a detailed exploration of RNA-based therapies, including categories such as ASOs, mRNA, and RNAi molecules, as well as their mechanisms of action. It also discusses distinct roles, mode of action, applications and major challenges for the above therapies, by which their molecular mechanisms are applied toward disease diagnosis and treatment in an accurate manner. The major challenges in bringing therapies from bench to bedside are being discussed. This review emphasizes on the present state of RNA-based drugs, highlighting the advanced innovations and breakthroughs, the challenges that still persists and need to be overcome and further developments at the therapeutic levels. It provides updated and crucial information on mechanism of action and clinical aspects of approved as well as under-trial RNA drugs. In addition, the paper reviews delivery systems and the stability of RNA based medicines, as well as potential progress in effectiveness and applications to different medical conditions. RNA-based therapeutics for treatment of cancer and infectious diseases are analysed, along with methods that contributes to safe and effective delivery of these drugs so as to fully explore the therapeutic potential of RNA-based drugs. The multifaceted role of RNA-based drugs in treatment of several diseases, major chemical modifications to be employed, various delivery systems that are utilized and challenges faced by RNA-based therapeutics in future are highlights of this review.

**FIGURE 1:**
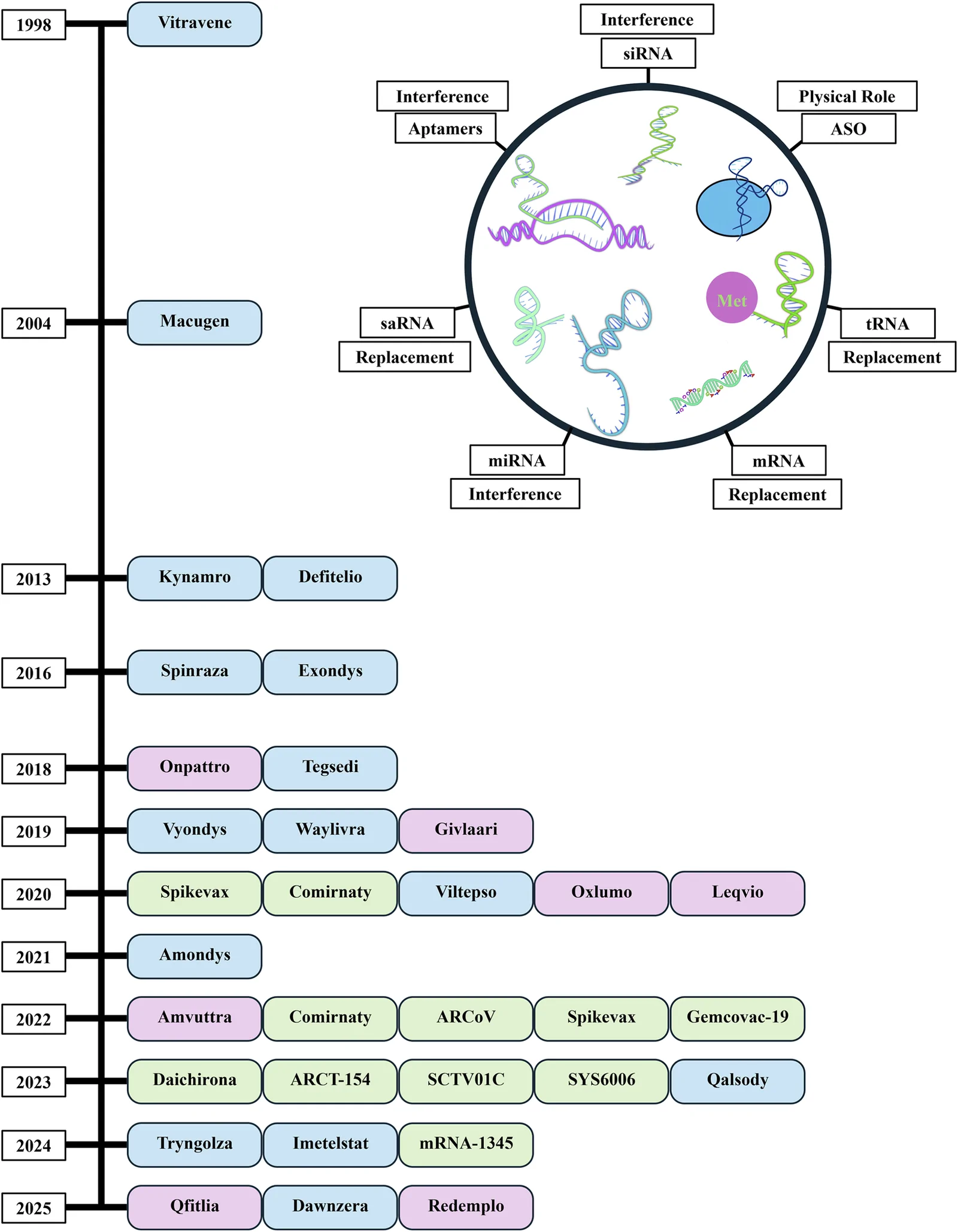
The advancement of RNA-based therapeutics is depicted in chronological way. Emerging developments in RNA therapeutics majorly include siRNA (small interfering RNA), ASO (Antisense oligonucleotides), tRNA (transfer RNA) replacement, RNA aptamers, miRNA (microRNAs) and saRNA (small activating RNAs) (Torrisi et al., 2025).

The therapeutic arena of RNA medicines covers three main modalities, all taking advantage of different mechanisms to provide clinical benefit. Of these strategies, ASOs constitute the most advanced platform, with more than four decades of research translating to many FDA-approved therapies (Figure 1). Single-stranded molecules that these are, they initiated sequence-specific gene silencing as a drug concept, and it is on this platform that contemporary RNA therapeutics have been established. Knowledge of how they have progressed from basic complementary sequences to complex, chemically-modified drugs offers valuable insight into the wider discipline of RNA medicine.

## 2 ASO therapeutics: fundamental principles and clinical applications

Synthetic RNA, DNA sequences or ASOs, are single stranded molecules made up of 20–30 ribonucleotides or deoxyribonucleotides. The goal of these carefully designed sequences is to bind to target RNA molecules by complementary base pairing, with a focus on the RNA of the gene of interest (Bajan and Hutvagner, 2020; Brentari et al., 2023). In 1978, Zamecnik and Stephenson showed that a chemically modified oligonucleotide could hybridize with its complementary sequence in a transcript of the Rous sarcoma virus. This stopped gene expression and viral replication, showing that ASOs could be used to treat diseases. This pioneering research employed a 13-nucleotide-long ASOs that was complementary to the target sequence of the Rous sarcoma virus, effectively hindering viral replication *in vitro* (Liang and Zhang, 2026). This ground-breaking finding initiated the use of ASOs as an effective and versatile instrument in molecular medicine. The first ASOs therapeutic, formivirsen, was subsequently approved for the treatment of cytomegalovirus retinitis, marking an important milestone in the clinical translation of antisense technology (Chaudhary et al., 2025).

The clinical success of approved ASOs extends beyond their ability to bind complementary RNA sequences. Modern ASOs incorporate chemical modifications such as phosphorothioate backbones and 2′-O-methoxyethyl (2′-MOE) sugar modifications (Lennox et al., 2025). These modifications enhance nuclease resistance, prolong tissue half-life, improve target affinity and reduce degradation *in vivo* (Ruchi et al., 2025). Successful ASO therapeutics generally target well-characterized disease-causing transcripts in which modulation of a single gene produces a measurable clinical benefit. Nusinersen (Spinraza) is a good example which, promotes inclusion of exon 7 in SMN2 pre-mRNA (Singh et al., 2026). Thereby restoring functional SMN protein levels in spinal muscular atrophy. Also, Tofersen (Qalsody) directly reduces mutant SOD1 mRNA levels in amyotrophic lateral sclerosis. These therapies demonstrate how precise target engagement, optimized chemistry and clear disease biology contribute to successful clinical translation (Rai et al., 2025).

The design and chemical alteration of ASOs have been essential in improving their stability and binding strength, significantly improving their efficacy as therapeutic agents Synthesized ASOs can target complementary sequences in pre-mRNA to influence the recruitment of splicing factors, thereby modulating splicing events. Furthermore, ASOs have an ability to form duplexes with mature mRNA resulting in preclusion of ribosome association at the site thereby inhibiting translation of protein. A second possible mechanism is the recruitment of RNase H to the target transcript for its degradation. This strategy is particularly beneficial for diseases in which the downregulation of expression of a toxic protein is therapeutic (for instance, some neurodegenerative diseases) (Bajan and Hutvagner, 2020). Here, ASO binding to target RNA take place followed by its cleavage through endogenous enzymes that causes downregulation of target transcripts (Lima et al., 2016). RNase H1 can act both on nuclear as well as cytoplasmic transcripts due to its ubiquitous nature causing cleavage of RNA-DNA geteroduplex (Curreri et al., 2023). ASOs have achieved remarkable progress in the treatment of various diseases, with several drugs now approved by the FDA. These ASO drugs belong to several chemical classes and act through distinct modes of action but share the same principle, since they are administered by different routes (Egli and Manoharan, 2023) (Table 1). The FDA-approved Fomivirsen is the first ASO to treat cytomegalovirus (CMV) retinitis in people with AIDS (Zhu et al., 2026). Another significant milestone in ASO therapy is Nusinersen, the first splice-correction ASO, approved for treating SMA (Signoria et al., 2023). In order to meet the immediate needs of patients and utilize the sequence-specificity of ASO therapies, personalized oligonucleotide therapies have been devised. Milasen is the first ASO that was designed for a specific patient, and it was made to treat neuronal ceroid lipofuscinosis type 7, a lethal neurodegenerative disorder (Wilton-Clark et al., 2025).

**TABLE 1:** Overview of FDA-approved ASOs and their clinical applications*.

Drug (brand)	Year	Mechanism of action	Route of delivery	Disease	References
Formivirsen (Vitavene)	1998	Binds to complementary sequences on the mRNA transcribed from the virus’s early transcriptional unit, thereby inhibiting replication of human CMV	Intravitreal	CMV retinitis	(Bege and Borbás, 2022)
Mipomersen (Kynamro)	2013	It binds to apolipoprotein B-100 mRNA, triggering its degradation by RNase H and thereby blocking protein translation	Subcutaneous	Homozygous familial hypercholesterolemia	(Chambergo-Michilot et al., 2022)
Nusinersen (Spinraza)	2016	By improving the splicing efficiency of survival motor neuron-2 (SMN2) pre-mRNA, it treats SMA by increasing the production of the SMN (survival motor neuron) protein	Intrathecal	SMA	(Wurster and Ludolph, 2018)
Eteplirsen (Exondys 51)	2016	Induces exon skipping in dystrophin pre-mRNA, restoring the reading frame of the Duchenne muscular dystrophy (DMD) gene to produce functional dystrophin	Intravenous	DMD	(Mendell et al., 2013)
Inotersen (Tegsedi)	2018	Inotersen degrades transthyretin mRNA, reducing serum transthyretin protein and tissue deposits	Subcutaneous	Familial amyloid polyneuropathy	(Gales, 2019)
Golodirsen (Vyondys 53)	2019	Golodirsen targets exon 53 of dystrophin pre-mRNA, promoting exon skipping to generate functional dystrophin and alleviate symptoms of Becker Muscular Dystrophy (BMD)	Intravenous	DMD	(Shimizu-Motohashi et al., 2016)
Volanesorsen* (Waylivra)	2019	Volanesorsen degrades apoC-III mRNA, preventing its translation and allowing triglyceride and chylomicron metabolism	Subcutaneous	Familial chylomicronemia syndrome	(Watts et al., 2020)
Viltolarsen (Viltepso)	2020	Viltolarsen binds exon 53 of DMD pre-mRNA, inducing exon skipping to restore the reading frame and produce a BMD-like dystrophin protein	Intravenous	DMD	(Watanabe et al., 2018)
Casimersen	2021	Casimersen targets exon 45 of DMD pre-mRNA, promoting exon skipping to generate a partially functional dystrophin protein and alleviate muscle tissue necrosis in DMD patients	Intravenous	DMD	(Shirley, 2021)
Tofersen (Qalsody)	2023	Targets SOD1 mRNA, reducing synthesis of toxic SOD1 protein	Intrathecal	SOD1-associated amyotrophic lateral sclerosis (ALS)	(Elman et al., 2026)
Imetelstat (Rytelo)	2024	It acts as a competitive inhibitor of telomerase enzymatic activity that induces apoptosis of malignant clones	Intravenous (IV) infusion	Low to intermediate-1 risk myelodysplastic syndromes (MDS) with transfusion-dependent anemia	(Thomas, 2025)
Olezarsen (Tryngolza)	2024	It decreases the production of apoC-III, a protein that regulates triglyceride metabolism in the blood	Subcutaneous	familial chylomicronemia syndrome	(Syed, 2025)
Donidalorsen (Dawnzera)	2025	Targets prekallikrein (KLKB1) mRNA, reducing bradykinin production and preventing hereditary angioedema attacks	Subcutaneous	Hereditary angioedema (HAE)	(Syed, 2026)

* Volanesorsen received EMA approval and represents an important clinically approved ASO therapeutic.

### 2.1 Modifications in ASOs

**FIGURE 2:**
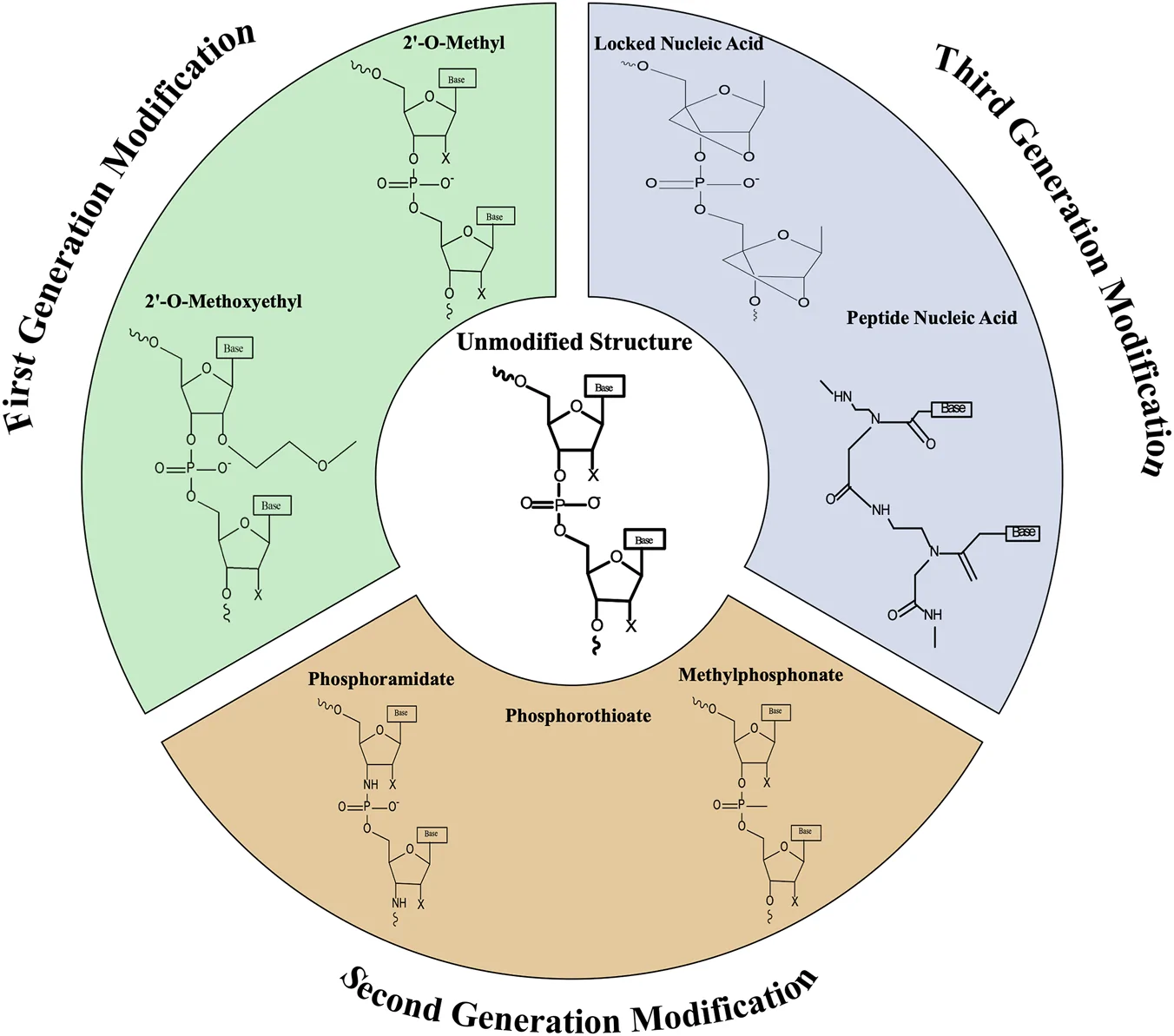
Antisense Nucleotide Modifications- The rapid breakdown of unmodified nucleic acids by nucleases renders them ineffective as therapeutic agents. To enhance the medicinal properties of ASOs, modifications are employed (Graczyk et al., 2023).

Native ASOs exhibit limited therapeutic utility because they are highly susceptible to nuclease-mediated degradation and possess poor cellular uptake. Their negatively charged phosphodiester backbone further restricts passive diffusion across biological membranes, resulting in reduced bioavailability and therapeutic efficacy (Sparmann and Vogel, 2023; Liang and Zhang, 2026). To overcome these limitations, a variety of chemical modifications have been introduced at the nucleobase, sugar, and backbone levels. These modifications enhance nuclease resistance, improve target-binding affinity and specificity, prolong circulation time, and reduce undesirable off-target effects. Consequently, successive generations of chemically modified ASOs have demonstrated markedly improved pharmacokinetic properties, stability, and therapeutic performance compared with their unmodified counterparts (Wang et al., 2025; Grijalvo et al., 2016) (Figure 2).

Modifications to the backbone structure characterize the first generation of ASOs (Karaki et al., 2019). Among these, phosphorothioate oligodeoxynucleotides (PS-ODNs) are the most commonly used first-generation ASOs. In PS-ODNs, a sulfur atom (PS), an amine group (phosphoramidates), or a methyl group (methyl phosphonates) takes the place of a non-bridging oxygen atom in the phosphodiester linkage (Beaman et al., 2014; Shen et al., 2019). Phosphorothioate-linked ASOs can bring in RNase H, which cuts the target RNA sequence. This modulation makes it harder for the kidneys to get rid of it, makes it stay in the body longer, makes it less likely to be broken down by nucleases, and lets it bind tightly to plasma proteins. However, first-generation ASOs still have problems, such as not getting enough cells to take them up, not being specific enough, and not having a strong enough binding affinity (Karaki et al., 2019). Second-generation ASOs have changes to the 2′ ribose sugar moiety, which leads to the creation of nucleosides that are restricted in their shape.

Two examples that have been studied a lot are 2′-O-Methyl (2′-OMe) and 2′-O-Methoxyethyl (2′-MOE) (Angrish and Khare, 2023). These modifications increase the tolerance of nucleases, increase target RNA binding affinity, and reduce toxicity of the drug by eliminating non-specific protein interactions (Lennox et al., 2025). Although these ASOs possess certain advantages, a major drawback is that they are not as effective at activating RNase H. The requirement of RNase H-mediated target RNA cleavage to increase antisense efficacy presents a significant challenge to many antisense strategies (Chiglintseva et al., 2025).

Third-generation ASOs have enabled the utilization of more complex chemical modifications, like phosphorodiamidate Morpholino Oligomers (PMOs). One PMO molecule features the backbone composed of morpholine rings connected by phosphorodiamidate linkages (Buthelezi et al., 2023). These changes give the drug better target affinity, better hybridization with mRNA, better stability against nucleases and peptidases, and better pharmacokinetic properties (Zhang and Zhang, 2023). There are many different types of third-generation nucleic acids being studied, such as ASOs, 2′-deoxy-2′-fluoro-β-D-arabino nucleic acids (FANAs), cyclohexene nucleic acids (CeNAs), and tricyclo-DNA (tcDNA). Peptide nucleic acid (PNA), locked nucleic acid (LNA), and phosphorodiamidate morpholino oligomer (PMO) remain the most extensively investigated due to their superior binding affinity, specificity, and biostability. These traits make them especially useful for diagnosis and treatment (Egli and Manoharan, 2023; Ge et al., 2006). Modifications are introduced in backbone structure that include PS linkages and PMOs in various ASO drugs that got FDA approval. Oxygen atoms replaced with sulphur renders stability by providing nuclease resistance, keeping RNase H activity intact. PS linkages further enhances protein interactions as albumin binding, hence improving pharmacological properties. Covalent binding of ASOs with antibodies and lipids represents another chemical modification to improve targeting to specific tissues. Attaching cholesterol or α-tocopherol to the 5′ end of therapeutic ASOs improves penetration through the blood-brain barrier upon intravenous injection.

### 2.2 Mode of action of ASO

ASOs are predominantly categorized into two principal types: those reliant on RNase H activity and those that are not, commonly known as steric block ASOs. Silencing ASOs operate by either promoting the degradation of complementary RNA molecules through RNase H-dependent pathways or by obstructing ribosomal function to inhibit the translation of the target gene via steric hindrance (Brentari et al., 2023). These ASOs can hybridize near the start codon to obstruct translation or bind to untranslated regions, thereby disrupting interactions with RNA-binding proteins. In addition to translational inhibition, ASOs can improve the translation efficiency of a target protein by inhibiting the translation of an upstream Open Reading Frame (ORF), thereby enhancing the expression of the intended protein. This makes the intended protein more likely to be expressed. ASOs may also modulate RNA splicing by interacting with splice sites or regulatory signals located within exonic or intronic sequences. This makes change splicing event occur with a very high accuracy (Bao et al., 2024).

**FIGURE 3:**
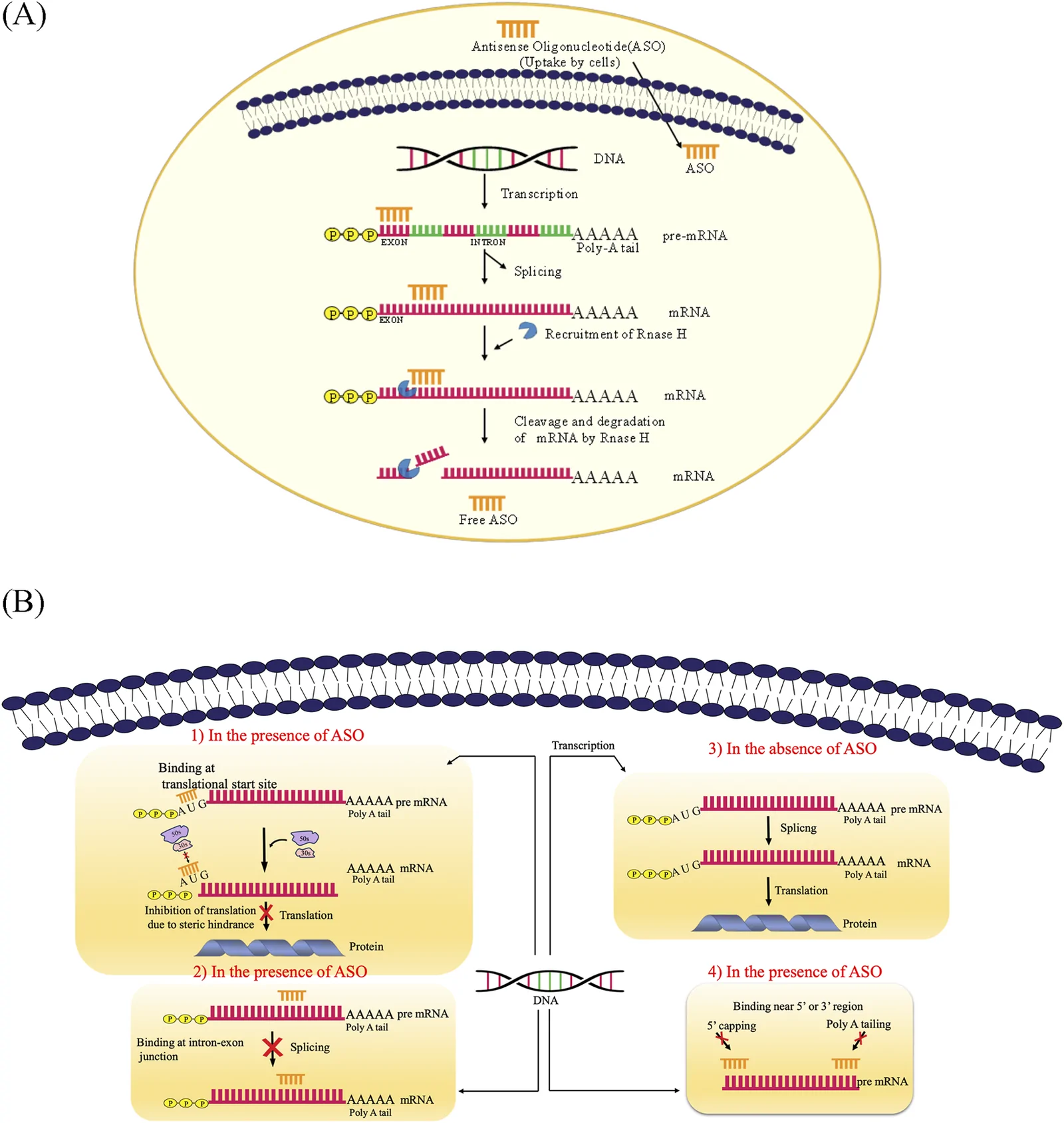
Mechanism of Action of ASOs: ASOs have an effect on cells after they get inside. The most common ways that this happens are through RNase H-mediated mRNA cleavage or through steric hindrance, which stops protein translation. **(A)** RNA cleavage mechanism: When an ASO binds to its target mRNA, it recruits RNase H, which degrades the mRNA, allowing the ASO to be reused for binding other mRNA molecules. **(B)** Occupancy-only mode of action: The physical obstruction caused by ASO binding to pre-mRNA/mRNA results in reduced protein synthesis. (1) ASO binding at the translation initiation codon, AUG, stops the target protein from being made by stopping ribosomes from attaching, which stops translation. (2) ASOs that attach to the intron-exon junctions of pre-mRNA can stop splicing by blocking the access of important splicing proteins through steric hindrance. This stops the formation of mature mRNA. (3) DNA is transcribed into pre-mRNA without ASOs. Then, splicing happens to make mature mRNA. Then, ribosomes attach to the start site of translation to make proteins that work. (4) ASOs that target the 5′ or 3′ ends of pre-mRNA can stop important changes that happen after transcription. Binding at the 5′ end stops capping, and binding at the 3′ end stops polyadenylation by stopping the proteins that are needed from coming in (Chakkyarath, 2025).

There are two main types of mechanisms that ASOs use to change RNA function: (i) RNA cleavage mechanisms, which cause the target mRNA to be cut by activating RNase H, and (ii) occupancy-mediated mechanisms, where the ASO binds to its target without cutting it (Angrish and Khare, 2023) (Figure 3).

#### 2.2.1 RNA cleavage mechanism

A widely used mechanism of action for ASOs is the activation of RNase H enzymes, which are ubiquitously expressed and account for the majority of antisense therapies currently being developed (Angrish and Khare, 2023). Mammalian cells possess two RNase H enzymes, RNase H1 and RNase H2, each of which specifically cleaves RNA within a DNA-RNA heteroduplex structure (Vickers and Crooke, 2014). Human RNase H1, in particular, has been shown to heavily impact activity for DNA-like ASOs. Changing the amount of RNase H1 expression, either up or down, has been shown to change DNA, as ASOs work in different cell lines, which directly indicates how crucial it is to their mode of action (Zinder and Lima, 2017). RNase H1 was found in different cellular compartments such as the nucleus, mitochondria, and cytoplasm by scientists (Schultz and Champoux, 2008). The enzyme attaches to the RNA-DNA heteroduplex using its N-terminal region as an RNA-binding domain. It cleaves the RNA strand 7–10 nucleotides from the 5′ terminus of the duplex, approximately one helical turn of the structure (Figure 3A) (Vickers and Crooke, 2014). RNase H-mediated mRNA degradation is fairly effective and is very effective at silencing target mRNA expression. This mode of action can lead to 80%–95% inhibition of both protein synthesis and mRNA levels, showing the effectiveness of RNase H-mediated ASOs for therapeutic action (Angrish and Khare, 2023). Since this process involves the activity of RNase H, which is active in both the nucleus and cytoplasm, these drugs can efficiently target noncoding elements.

#### 2.2.2 Occupancy-only mechanism

High-affinity ASOs are employed in steric block or occupancy-only mediated mechanisms to bind target RNA without inducing its degradation. These ASOs do not directly degrade RNA; rather, they modify gene expression through diverse mechanisms. The underlying mechanism is based on the classical concept of competitive inhibition, in which an antagonist blocks the attachment of a natural activator, thereby modifying the biological response. Analogously, ASOs bind to their target mRNA sequences, preventing interactions with key proteins and factors essential for mRNA processing. The splicing of pre-mRNA, which involves the removal of non-coding sections (introns) to create mature mRNA, is an essential stage in mRNA intermediate metabolism. Proteins and tiny nuclear RNAs must work together in the spliceosome complex to accomplish this extremely sequence-specific splicing process. Targeting sequences close to the intron/exon boundary can cause ASOs to block the required cleavage processes, which prevents crucial splicing factors from recognizing them. This interference ultimately suppresses protein translation (Figure 3B). This kind of mechanism is especially useful for treating diseases caused by improper mRNA splicing. ASOs can stop the production of functional proteins linked to disease by changing splicing (Mansoor and Melendez, 2008; Crooke, 2017). Although ASOs proved the concept of RNA targeting for therapy, their direct hybridization-based mechanism had inherent limitations in potency and cell delivery (Xue et al., 2018). The discovery of RNA interference in the late 1990s transformed gene regulation knowledge by unveiling an endogenous cellular machinery with the capability to achieve considerably more effective gene silencing than the conventional antisense strategy. This enzymatic amplification system, evolved spontaneously for viral protection and gene control, provided pharmaceutical scientists with a more potent armoury for therapeutic intervention. The shift from engineered antisense molecules to leveraging cellular RNAi machinery was a landmark change in RNA therapeutic innovation.

## 3 siRNA and miRNA as therapeutic approach

**FIGURE 4:**
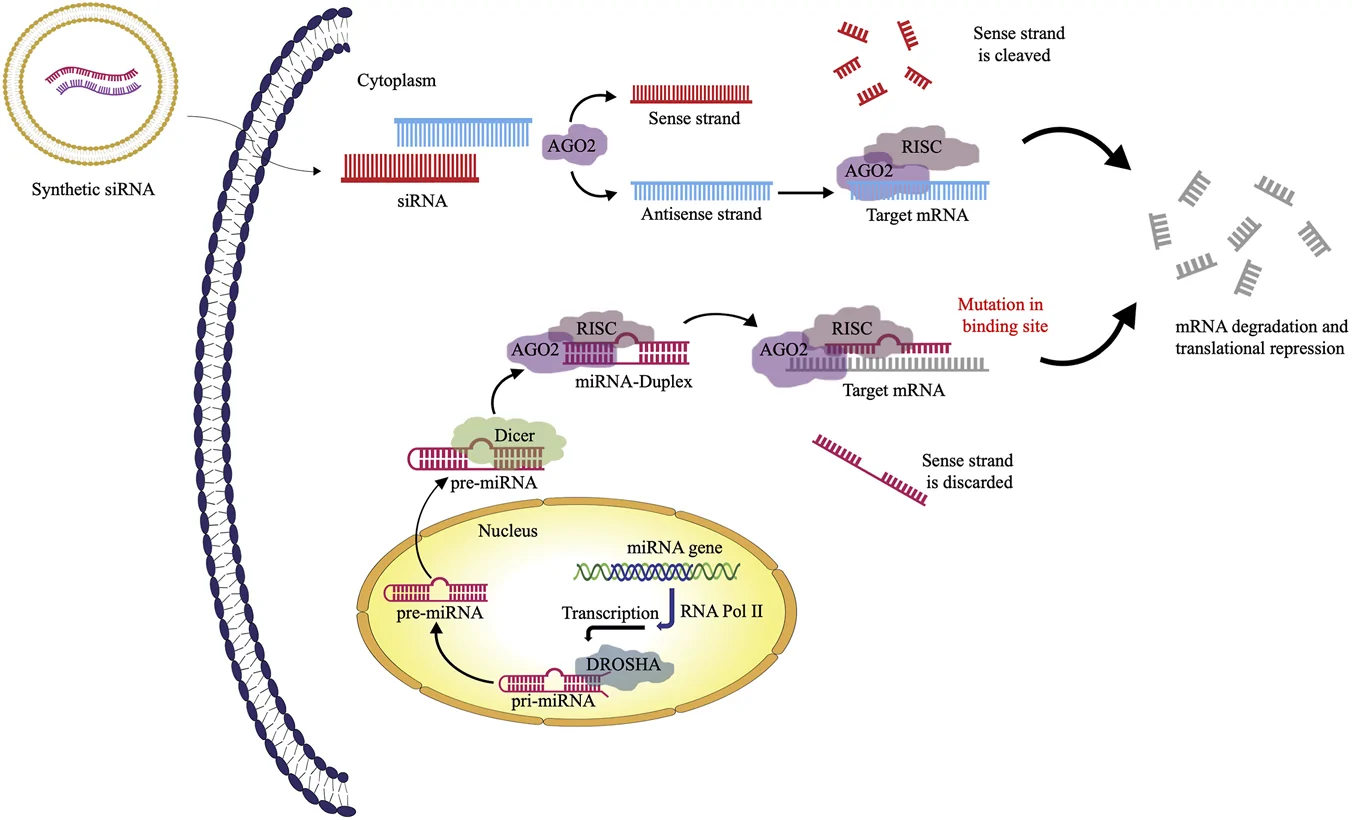
Schematic representation of siRNA and miRNA in RNA therapeutics. This figure illustrates the mechanisms by which siRNA and miRNA modulate gene expression in RNA therapeutics.

Andrew Fire and Craig Mello discovered in 1998 that double-stranded RNA (dsRNA) was the cause of RNAi in *Caenorhabditis elegans* (Fire et al., 1998). Their results went against what was then known about how antisense RNAs worked, which was that they only worked by directly binding to target mRNA and blocking its expression. Their research uncovered the presence of an enzymatic silencing mechanism. Dicer, a cytoplasmic RNase III enzyme, processes double-stranded RNAs into siRNAs or miRNAs (Figure 4). This can happen when the RNAs are brought into the cell from the outside or made inside the cell as precursor RNAs with stem-loop or short hairpin structures (Sparmann and Vogel, 2023; Valencia-Sanchez et al., 2006). siRNA, a double-stranded oligonucleotide typically comprising 19–25 base pairs, employs RNAi technology to effectively suppress specific gene expression via complementary-based cleavage of mRNA (Zhao et al., 2022; Qiu et al., 2016). The exploitation of endogenous RNAi pathway is done to cause modulation of target RNA expression by siRNAs (Kang et al., 2023). siRNA binds to the RISC to help break down target mRNA sequences. During this process, siRNA is split into a sense strand and an antisense strand. The sense strand is thrown away, and the antisense strand pairs with the mRNA sequence that is the opposite of it. The antisense strand, with help from the endonuclease Argonaute 2 (AGO2), directs the cleavage of the target mRNA, which leads to the breakdown of the transcript and stops translation (Figure 3A) (Kole et al., 2012; Bajan and Hutvagner, 2020). Till now, six siRNA-based drugs have got approval from FDA. These drugs are patisiran, givosiran, lumasiran, Vutrisiran, Inclisiran and Nedosiran (siRNA drugs have siran as suffix to their name) (Xiao et al., 2025). Endogenous miRNAs are small RNA molecules, 21 to 23 nucleotides long, that come from a two-step maturation process that uses the Drosha and Dicer complexes. These miRNAs regulate gene expression via the RNA-induced silencing complex (Brentari et al., 2023). Unlike siRNAs, miRNAs typically interact with partially complementary target sites, leading to translational repression and transcript degradation. miRNAs are important regulators of gene expression that affect many bodily functions, including those that are related to diseases. Because they can control gene expression, miRNAs are promising therapeutic targets for a number of conditions while miRNA degradation is being considered as a significantly potential therapeutic agent (Rupaimoole and Slack, 2017).

miRNA mimics and antimiRs are the two types of miRNA-based therapies. Synthetic double-stranded oligonucleotides called miRNA mimics are made to mimic the actions of naturally occurring miRNAs and work similarly to siRNAs. These mimics can restore the function of dysregulated miRNAs (Dammes and Peer, 2020; Sparmann and Vogel, 2023). AntimiRs, however, are structurally similar to ASOs and specifically bind to the mature strand of a given miRNA, thus blocking its function (Sparmann and Vogel, 2023). Single miRNA can associate with target miRNAs even when the binding is not perfect, hence can alter the expression of range of protein-coding RNAs. Thus, each miRNA can potentially modulate multiple targets involved in similar processes, generating amplified response. Despite significant development and progress in preclinical research, only few miRNA-based therapeutics are undergoing clinical advancements due to toxicity issues. Till date not even a single miRNA drug has reached phase III trial with no approval by FDA (Table 2). INT-IB3 (InteRNA Technologies) is a synthetic miRNA that mimics endogenous tumor suppressor miR-193a-3P. INT-IB3 execute multi-target mechanism for tumor suppression which involves anti-metastatic, anti-proliferative, anti-migration, apoptosis induction and modulation of tumor microenvironment (Liu et al., 2025). It works by down regulating oncogenic signalling pathways and upregulation of tumor-suppressive PTEN pathway (Su et al., 2024). Though Phase 1 trial indicated INT-IB3 as effective against solid tumors, the study was terminated due to insufficient findings. Recently there has been introduction of diet-derived exogenous miRNAs or xenomiRs which can modulate gene expression and downstream biological functions by moving through circulatory system and tissues. The deeper evaluation of miRNA therapeutics is required for enhancement of their specificity, selectivity and targeted delivery, along with reduction in side-effects (Diener et al., 2022).

**TABLE 2:** Representative miRNA-targeted therapeutics in clinical and translational development.

Disease	Target miRNA	Therapeutic candidate/Molecule	Brand/Company	Development status (2026)
Alport nephropathy/Kidney fibrosis	miR-21	Lademirsen (RG-012)	Regulus therapeutics and Genzyme	Clinical development discontinued after Phase II
Hepatitis C virus infection	miR-122	Miravirsen (SPC3649)	Santaris Pharma/Roche	Completed early clinical studies; development not advanced further
Hepatitis C virus infection	miR-122	RG-101	Regulus Therapeutics	Clinical development terminated because of safety concerns

Despite extensive preclinical success miRNA therapeutics have encountered significant obstacles during clinical translation. Unlike siRNAs, which are designed to silence a single transcript through perfect sequence complementarity. Individual miRNAs can regulate hundreds of genes simultaneously. While this broad regulatory capacity may provide therapeutic advantages. It also increases the likelihood of unintended pathway modulation, off-target effects and toxicity. The challenges associated with miRNA therapy were exemplified by MRX34, a liposomal miR-34a mimic developed for cancer treatment (Generalov et al., 2026). Although promising antitumor activity was observed in preclinical models, the phase I clinical trial was terminated due to severe immune-related adverse events. Several anti-miRNA programs have demonstrated encouraging biological activity but failed to achieve an adequate balance between efficacy and safety. These findings indicate that improved tissue-specific delivery systems and enhanced control of off-target effects remain essential for the successful clinical development of miRNA therapeutics (Tuteja et al., 2026).

Patisiran became the first clinically approved RNA interference (RNAi)-based therapeutic in 2018, marking a major milestone in the development of siRNA medicines. Since then, several additional siRNA therapeutics have entered clinical practice (Traber and Yu, 2024) (Table 3).

**TABLE 3:** FDA - approved siRNA drugs: mechanism of action, delivery methods and targeted disease.

Product	Mechanism of action	Route of delivery	Disease	References
Patisiran (Onpattro)	Processed by Dicer, binds to the RNA-induced silencing complex (RISC), which unwinds one of the two strands bound strand targets and cleaves transthyretin mRNA, rendering it non-functional and allowing repeated mRNA destruction	Intravenous	Polyneuropathy of hereditary transthyretin-mediated amyloidosis	(Maurer et al., 2023)
Givosiran (Givlaari)	Givosiran, taken up by hepatocytes via asialoglycoprotein receptors, is processed by RISC to cleave 5-aminolevulinic acid synthase (ALAS1) mRNA, preventing ALAS1 enzyme synthesis and reducing neurotoxic heme intermediates	Subcutaneous	Acute hepatic porphyria	(Syed, 2021; Petrides, 2026)
Lumasiran (Oxlumo)	Targets and silences hydroxyacid oxidase 1 (HOA1) mRNA to reduce glycolate oxidase levels, decreasing glyoxylate and thus oxalate production, For the treatment of primary hyperoxaluria type 1	Subcutaneous	Primary Hyperoxaluria Type 1	D’ambrosio and Ferraro, 2022
Inclisiran (Leqvio)	Inclisiran, taken up by hepatocytes, binds to RISC to cleave Proprotein convertase subtilisin-kexin type 9 (PCSK9) mRNA, reducing PCSK9 production and increasing Low-density lipoprotein (LDL) receptor recycling for enhanced LDL-cholesterol (LDL-C) uptake	Subcutaneous	Atherosclerotic Cardiovascular Diseases	(Harbi, 2025)
Vutrisiran (Amvuttra)	It works in the similar way as Patisiran but employs Alnylam’s improved stabilization chemistry and uses GalNAc-siRNA delivery platform	Subcutaneous	Hereditary transthyretin-mediated amyloidosis (hATTR)	(Fontana et al., 2025)
Nedosiran (Rivfloza)	This drug halts the expression of liver lactate dehydrogenase (LDH), which converts glyoxylate into oxalate in hepatic cells. Hence, it decreases the overproduction of oxalates	Subcutaneous	Primary hyperxaluria type 1	(France and Syed, 2025)
Fitusiran (Qfitlia)	Targets antithrombin (SERPINC1) mRNA, lowering antithrombin levels and restoring thrombin generation to improve hemostasis	Subcutaneous (GalNAc-conjugated)	Hemophilia A and B with or without inhibitors	(Haider et al., 2026)
Plozasiran (Redemplo)	Targets apolipoprotein C-III (APOC3) mRNA, reducing plasma triglycerides and improving lipid	Subcutaneous (GalNAc-conjugated)	Familial chylomicronemia syndrome (FCS)	(Yin et al., 2026)

The development of miRNA therapeutics has yet to result in any approved drug candidates for clinical use. Currently, multiple miRNA molecules are undergoing clinical trials; but none have progressed to Phase 3 trials (Seyhan, 2024).

The clinical success of approved siRNA therapeutics is largely attributable to advances in targeted delivery and RNA stabilization (Tam et al., 2013). Early RNA interference approaches were limited by rapid degradation and inefficient cellular uptake. These challenges were overcome through the development of LNP systems and GalNAc conjugates. LNP and GalNac facilitate receptor mediated uptake by hepatocytes through the asialoglycoprotein receptor (Qin et al., 2025). Once internalized the antisense strand is incorporated into the RISC complex. RISC catalytically cleaves complementary target mRNA molecules enabling prolonged gene silencing. Patisiran is the first approved siRNA therapeutic, demonstrated that efficient hepatic delivery could achieve substantial transthyretin suppression and clinical benefit in hereditary transthyretin amyloidosis (Porcari and Fontana, 2026; Pratt and MacRae, 2009). Subsequent agents such as inclisiran, vutrisiran, fitusiran and plozasiran further validated this platform by combining potent target knockdown with infrequent dosing schedules and favourable safety profiles. The most successful siRNA therapeutics target liver-expressed genes associated with monogenic or metabolically well-defined disorders by maximizing efficacy while minimizing off-target toxicity (Kumari et al., 2023).

The achievements of ASOs and RNA interference therapies proved the therapeutic potential of gene silencing, but these methods remain inherently restricted to suppressing or eradicating protein expression. With the discovery came a revolution: the understanding that RNA was not only a candidate for inhibition, but also a vector for protein delivery itself. Instead of suppressing troublesome genes, mRNA therapies allow cells to produce useful proteins, essentially turning the patient’s own cells into factories for drugs. This theoretical flip RNA from target to medicine has created therapeutic avenues for long-intractable conditions and, in essence, broadened the reach of what RNA-based treatments can do.

## 4 Using mRNA as a therapeutic methods

In recent years, mRNA-based therapies have become a revolutionary area of medicine, changing gene therapy by making vaccines, immunomodulatory agents, and anticancer therapies (Hobernik and Bros, 2018). mRNA is a ssRNA transcribed from DNA. It serves as a carrier of coding information essential for protein synthesis, which is subsequently translated into functional proteins (Metkar et al., 2024; Qin et al., 2022).

*In vitro* transcription (IVT) is a common way to make synthetic mRNA in labs. It uses a DNA plasmid and bacteriophage RNA polymerase. This method makes mRNA molecules that are structurally similar to those that cells make on their own. These mRNA molecules have important parts like a 5′ cap, a 5′ untranslated region (UTR), an open reading frame (ORF), a 3′ UTR, and a poly(A) tail. These structural elements are critical to the stability and the translation of mRNA, which play a big role in how well mRNA-based drugs work. Once synthesized, mRNA is subjected to a rigorous filtering process that removes things like reactants, partial transcripts and all sorts of other components that should not be there. This purification is very important to lower the chances of immune-stimulating effects (Żak and Zangi, 2025).

**FIGURE 5:**
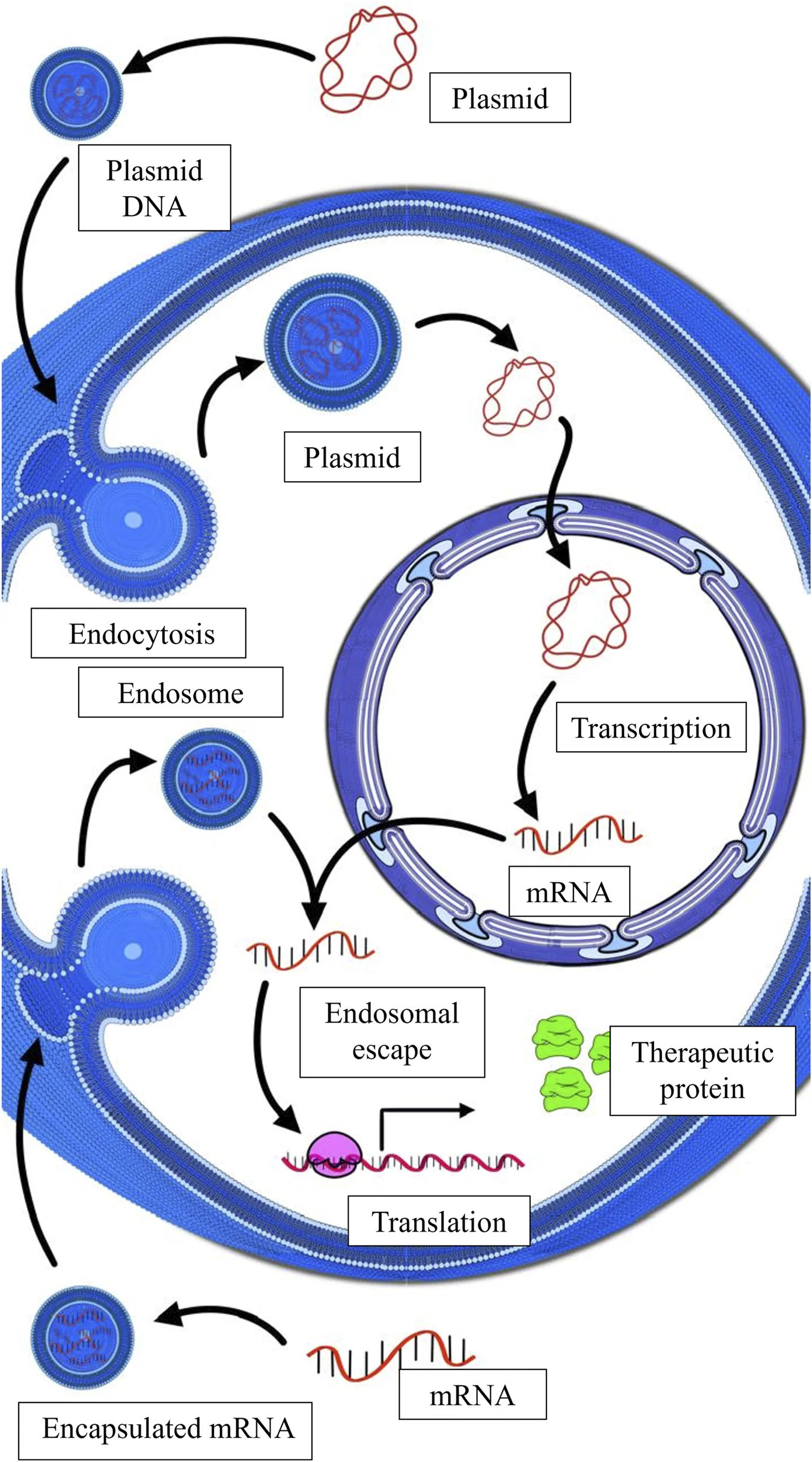
mRNA exhibits greater transfection efficiency and reduced toxicity, compared to DNA-based therapies as mRNA is not required to regain entry into nucleus to integrate itself into genome to execute its function. Furthermore, mRNA has led to development of mRNA-based vaccines and mRNA replacement treatments for multiple diseases (Gentry et al., 2023).

mRNA shows a higher transfection efficiency and lower toxicity than DNA-based therapies. This is mainly because the mRNA does not need to regain entry into the nucleus to merge into genome in order to fulfil its function (Wang et al., 2023). Indeed, because it is so crucial for translating the genetic information in DNA to proteins and acts as a bridge between the central dogma of molecular biology, mRNA has given rise to many new approaches to treat illnesses like mRNA-based vaccines and mRNA replacement treatments (Zhang et al., 2019; Camperi et al., 2025) (Figure 5).

Various approaches can be used for the development of mRNA-based therapies, such as rectification of defective gene expression, therapeutic protein manufacturing, generating immunity by vaccination with specific antigen proteins produced by translation of modified mRNA (Li J. et al., 2025), cell therapy upon *ex-vivo* transfection of mRNA (Volodina and Smirnikhina, 2025) and *in vivo* expression of therapeutic antibodies or enzymes (Żak and Zangi, 2025).

### 4.1 mRNA vaccines

The first successful application of mRNA as a vaccine platform was documented in 1993 after it was demonstrated that NP-specific cytotoxic T-cell responses may be induced by subcutaneous delivery of liposome-encapsulated mRNA expressing the influenza virus nucleoprotein (Martinon et al., 1993). mRNA molecules that encode adjuvants and/or antigens function as vaccines by either stimulating the immune system to combat cancer (therapeutic vaccines) or eliciting protective immunity against infectious illnesses (prophylactic vaccinations) (Lavelle and Mcentee, 2024; Iavarone et al., 2017). The clinical success of mRNA vaccines during the COVID-19 pandemic demonstrated the therapeutic potential of mRNA technology on an unprecedented scale. BNT162b2 and mRNA-1273 were among the first mRNA vaccines to achieve widespread clinical use, establishing mRNA as a versatile platform for both infectious disease prevention and future therapeutic applications (Teo, 2022; Chaudhary et al., 2021) (Table 4). Both humoral and cellular immunity are boosted by mRNA vaccines (Kramps and Probst, 2013).

**TABLE 4:** FDA-approved mRNA vaccines: delivery methods and disease indications.

Product	Mechanism of action	Route of delivery	Disease	References
Tozinameran (BNT162b2)	Has lipid nanoparticles (LNPs) that contain nucleoside-modified mRNA. These nanoparticles are then carried into host cells to create a modified SARS-CoV-2 spike protein, which activates cellular immune responses and neutralizing antibodies to defend against infection	Intramuscular	COVID-19	(Valnet-Rabier et al., 2023; Jackson et al., 2020)
mRNA-1273	Uses LNPs encapsulated synthetic mRNA to encode the SARS-CoV-2 spike protein, which causes human cells to produce the antigen and start an immune response to protect against the virus	Intramuscular	COVID-19	(Teo, 2022)
mRNA-1345 (mRESVIA)	LNP-encapsulated mRNA encoding the respiratory syncytial virus (RSV) prefusion F glycoprotein, stimulating protective immunity against RSV infection	Intramuscular	Prevention of RSV lower respiratory tract disease in adults	(Tregoning, 2024)
ARCT-154 (KOSTAIVE)	Self-amplifying RNA (saRNA) vaccine encoding the SARS-CoV-2 spike protein. The replicon component enables intracellular RNA amplification, resulting in prolonged antigen expression at lower doses compared with conventional mRNA vaccines	Intramuscular	COVID-19	(Okechukwu Paul-Chima et al., 2026)

mRNA controls how antigens reach the target sites of the cell, either naturally or through engineering. These vaccines instruct the cells in the body to produce the antigen that the mRNA is encoding. Optimizing the mRNA sequence reduces the body’s natural immune responses and boosts antigen expression in host cells.

When host cells translate mRNA, the resulting antigen expression is like a pseudo infection that is similar to the real pathogen. This triggers strong cellular and humoral immune responses (Heine et al., 2021; Sushnitha et al., 2020).

**FIGURE 6:**
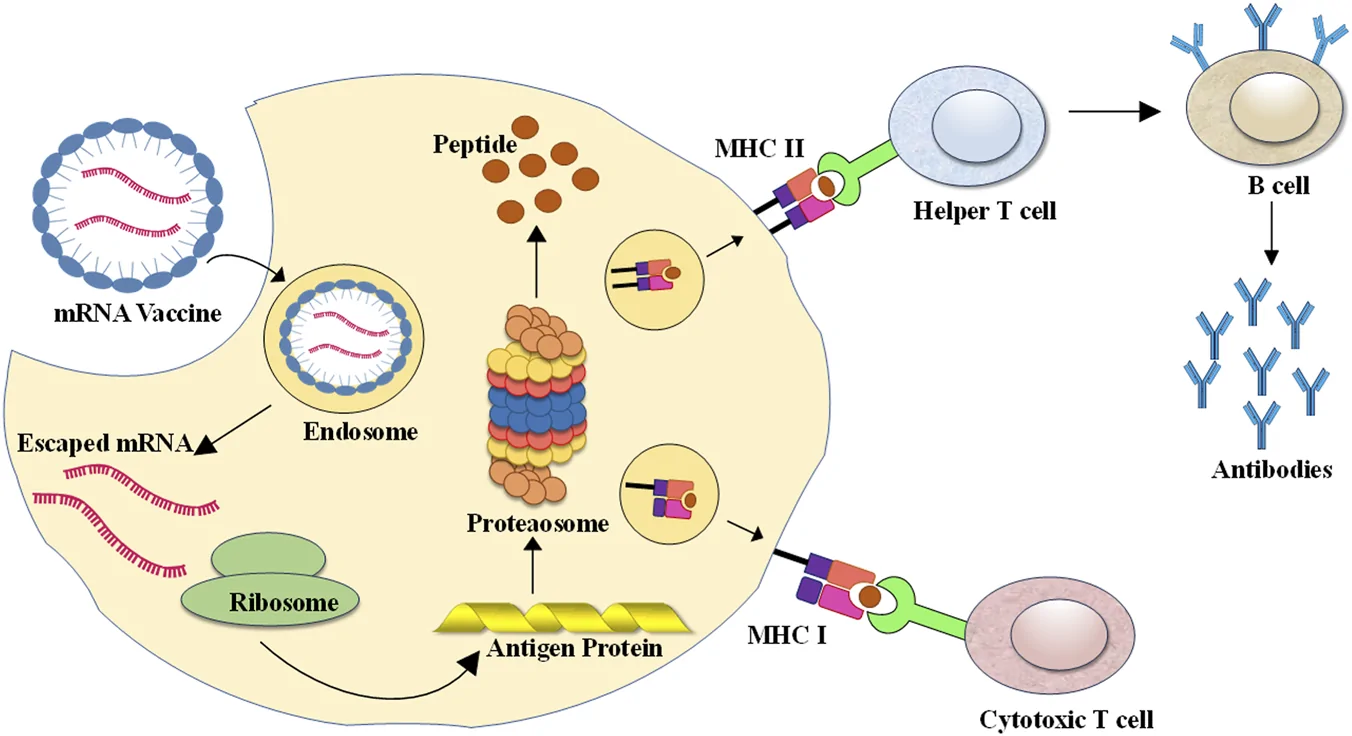
The figure illustrates how mRNA-based vaccines induce an immune response. The method starts with the administration of synthetic mRNA which encodes the antigen of interest usually packaged into lipid nanoparticles (LNPs) and directed into host cells. Once inside the cytoplasm, the host’s ribosomes transcribe the mRNA into the target protein, or antigen. The major histocompatibility complex (MHC) molecules are used to process and display the generated protein on the cell surface. This display triggers the activation of antigen-specific T cells, initiating a strong immune response. Concurrently, the antigen is identified by B cells, resulting in their activation and subsequent differentiation into plasma cells that produce neutralizing antibodies. The interplay between cellular and humoral immune responses facilitates the formation of immunological memory, thereby providing enduring protection against the pathogen (Verbeke et al., 2022).

Based on the type of mRNA used, vaccines are categorized into conventional mRNA vaccines and self-amplifying RNA (saRNA) vaccines. Conventional mRNA vaccines resemble host mRNA and encode only the antigenic material, while saRNA vaccines include a replicase enzyme that amplifies the RNA during transcription. Self-amplifying mRNA replicates the behavior of single-stranded positive-sense RNA viruses, enhancing the duration and intensity of antigen expression (Papukashvili et al., 2022). This means that a smaller dose of the vaccine can keep the immune system active for a longer time and make more antigens, which makes the vaccine more effective. Also, self-amplifying mRNA can encode more than one antigen in a single replicon, which makes the vaccine even stronger (Figure 6). When the purified RNA gets into the host cell, RNA polymerase drives immense translation and replication (Wang et al., 2021; Casmil et al., 2025).

### 4.2 mRNA protein replacement therapy

Protein replacement therapies work great in treating diseases that are caused by mutations or the absence of certain proteins. These treatments are often used to help people with metabolic disorders, lysosomal storage diseases, and blood disorders (Żak and Zangi, 2025). mRNA-based protein replacement therapy is a new development in this field that offers an innovative way to tackle insufficient protein in the body (Vavilis et al., 2023). This treatment involves sending mRNA molecules that code for the missing protein into the patient’s body, which allows the patient’s cells to synthesize the required protein (Sahin et al., 2014; Crunkhorn, 2013).

Researchers have mainly concentrated on mRNA-based protein replacement therapy for rare monogenic disorders. These conditions arise from single-gene mutations that disrupt or eliminate the synthesis of functional proteins encoded in the human genome (Gorzelany and de Souza, 2013). The diseases caused by genetic defects and inborn errors of metabolism needs to be addressed by their cause. mRNA is being employed for production/encoding of peptides and proteins to restore their deficiencies through protein replacement therapies. When proteins are defective or missing, the administration of required protein is primary approach to be applied. However, short half-life of administered proteins, structural instability and ability to elicit immunogenic responses tend to act as hurdles for therapeutic purposes. The mRNA-based protein replacement approach renders multiple advantages over other protein or DNA-based methods. It offers increased safety as there is no integration to host genome, the transient nature of RNA reduces the possibility of mutations. Further, modified mRNA (modRNA) technology, which eliminates mRNA recognition by TLRs, can be utilized so as to prevent intracellular cleavage of mRNA by RNase that can generate immune response, eventually causing cytokine-induced toxicity (Bitounis et al., 2024). The success of mRNA vaccines in fighting COVID-19 has made this technology very popular. It shows how quickly and easily it can be developed and used in other mRNA-based medicines (Anand and Stahel, 2021). This platform is very flexible; thus, it can make almost any protein. This is why it is used to treat a wide range of diseases, including genetic disorders, cancers, and infections (Schlake et al., 2012).

There are a lot of problems with putting mRNA protein replacement therapy into practice, even though it has a lot of potential. The most important thing is to make delivery systems that are safe and work well so that mRNA molecules can reach their target cells and work without causing immune responses (Liang et al., 2021). The mRNA platform has been useful for making vaccines, but it is still being explored for the treatment of other medical conditions, such as rare hereditary disorders, where it could be used to replace proteins (Chien et al., 2026).

## 5 Circular RNA as therapeutic agent

Circular RNAs (circRNA) represent a new group of non-coding RNA that consists of a covalently closed-loop structure, which are more stable than linear RNAs formed during a unique form of alternative splicing known as backsplicing where joining of downstream splice donor site happens with upstream splice acceptor site (Hwang and Kim, 2024; Memczak et al., 2013). CircRNA were first identified in the year 1976 in an electron microscopy-based study of RNA viruses (Lee and Koonin, 2022). CircRNA was later successively identified in humans, mice, rats such as yeast mitochondria, human E-twenty-six-1 (ETS-1) gene, human P450 2C18 gene, rat cytochrome P450 2C24 gene and hepatitis delta virus (HDV) (Jeck and Sharpless, 2014). CircRNA are formed of a covalently closed loop structure without 5’caps and 3’tails. Per host gene only 3–10 different circRNA isoforms are formed thus resulting in tens of thousands of distinct circRNA per cell type (Ebbesen et al., 2017; Santer et al., 2019). In circRNA, majority are 3′-5’linked circles which contains only exons and not intervening introns. On an average, circRNA consists of around 1–5 exons and are 500 ribonucleotides (nts) long (Liu and Chen, 2022). High-throughput RNA sequencing has revealed that at least 20% of currently active genes produce circRNAs (Zhang and Zhao, 2025). In 20% of all processes, introns actually retain a place in the mature circRNA, forming the class of Exon-Intron-containing-circular-RNAs (ElciRNAs). A much smaller percentage of circRNAs consist solely of introns, but not exons, circular intronic RNAs (ciRNAs), which are not a product of backsplicing, but of traditionally sliced-out intronic lariats. ElciRNAs and ciRNAs are characterized by their nuclear presence and activity (Yang J. et al., 2025; Li et al., 2015). However, circRNAs has been discovered for a long period of time but were considered a by-product of mis-splicing. Because of their structural specificity, low abundance and unknown functions, they were considered ancient and were treated as a genetic accident or an operational error (Liu et al., 2021). Recently, CircRNAs are starting to emerge as a heterogeneous group of molecules implicated in the modulation of gene expression through transcription regulation, protein and miRNA activity (Han et al., 2018). CircRNA expression is tissue and cell type specific and can often be independent of the linear host gene expression level, suggesting that regulation of expression could be a crucial feature in terms of CircRNA function control (Misir et al., 2022).

### 5.1 CircRNAs mode of action

**FIGURE 7:**
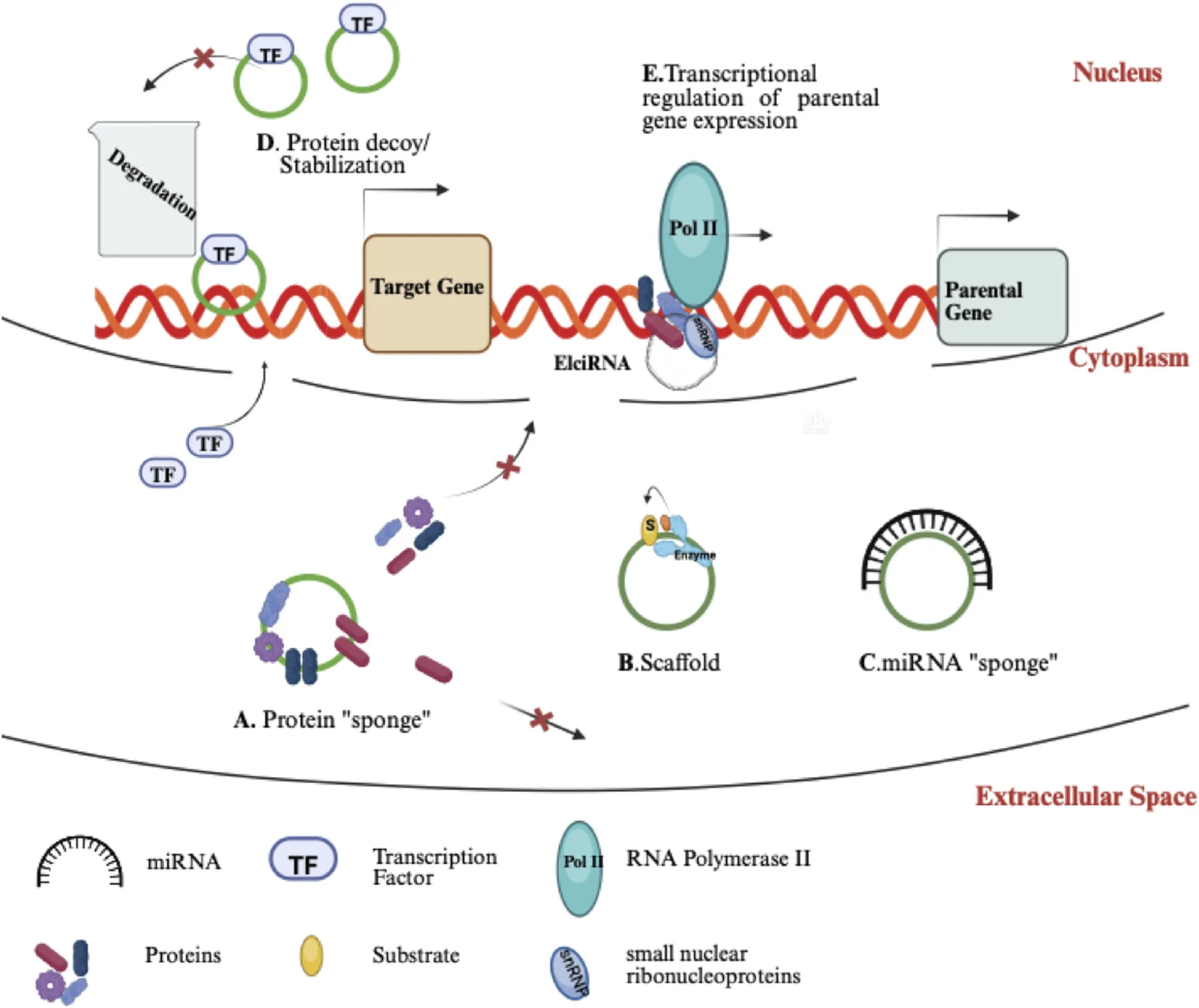
This figure illustrates the working of circRNAs. CircRNAs can act as (A) Protein “sponge” (B) Scaffold (C) miRNA “sponge” and (D) Protein decoy/stabilization (Liu and Chen, 2022).

After the large scale RNA-seq studies in 2012 lead to systematic search for circRNA functions. Few studies revealed that circRNA CDR1as was acting as a competing RNA molecule by sponging miRNA (Singh et al., 2024). Though most circRNA do not contain multiple binding sites thus miRNA is just one of its many potential functions. Many other molecular mechanisms for circRNA were revealed later on such as sponging of proteins, modulation of transcription and splicing and acting as scaffolds for protein complexes (Gu et al., 2023) (Figure 7).

#### 5.1.1 Different activities of CircRNA

miRNA belongs to the class of small RNAs having size of approximately 20 nucleotides. The most frequently observed activity from a circRNA is sponge activity. These circRNA contains multiple binding sites for miRNA within their nucleotides sequence, therefore they can bind to miRNA which prevents them from binding to their canonical mRNA target genes thus sponge effect. For example, circRNA CDR1as contains around 70 miRNA sites for miR-7 (Cerda-Jara et al., 2026).

Not long after the discovery of sponge activity of circRNA, other molecular functions were also found and explained. Shortly after sponge activity, it was shown that circ-Mbl contains several binding sites for Mbl protein and that circ-Mbl and MBL protein biogenesis is highly regulated by the transcriptional products as well as MBL protein from the mbl locus itself (Shi et al., 2022).

Circ-Amotl1 is a tumorigenic circRNA that functions in the nucleus, whereas circ-Foxo3 is inversely correlated with cell proliferation and functions as a protein-binding partner in the cytoplasm. Circ-Amotl1, which is extensively expressed in human cancer cell lines and patient tumour tissues, interacts with the oncogenic transcription factor c-Myc to cause tumorigenicity (Yang et al., 2017; Yang S. et al., 2025). By binding and keeping c-Myc in the nucleus, stabilising c-Myc, and upregulating c-Myc target genes, circ-Amotl1 can promote cell division, decrease apoptosis, and produce a highly carcinogenic phenotype. This study provides an illustration of how a circRNA could stabilise and decoy individual proteins to a certain cellular compartment (Luo et al., 2019).

The precisely designed and engineered circular RNA can destroy pathogenic and abberant RNA secondary structures by exploiting competitive annealing, thus inhibiting the synthesis of defective proteins and restoring normal RNA functioning. The advantages of circular RNA as therapeutics agents are conferred by circularization chemistry, structure design and delivery formulations (Guo et al., 2014). The field of circular RNA therapies tends to be promising next-generation of RNA-based drugs that provides enhanced stability with lowered immunogenicity (Holdt et al., 2018). Currently, no circRNA drugs have been approved by FDA, yet developments in their delivery method and clinical studies can lead to progression of circRNA in coming years. The advancement of understanding of the role of circRNAs in CNS pathologies and for maintaining normal brain function has been a key research area which depicts role of circular RNA in Parkinson’s disease, Alzheimer’s disease and stroke (Arruri et al., 2026). The expanding landscape of circRNA therapeutics in chronic diseases, oncology and immunology makes it a desirable choice in future therapeutics as they appear to be promising candidates in near future. Table 5 indicates circRNA as playing indispensable role in regulating pathophysiology of various diseases, particularly in the fields of oncology. Further, circular RNA also plays major role as clinical biomarkers for diagnosis of several diseases as Cardiovascular diseases due to their disease and tissue-specific expression patterns (Chen et al., 2026). Currently, endless RNA (eRNA) has been developed by a new biotech company Laronde Bio. That involves a circular shaped structure which can easily undergo translation. This Endless RNAs possess an internal ribosomal entry site or IRES using which ribosomes associate with circular RNA for translation and *de novo* protein synthesis persists for long in loop due to circular structure. The circular structure renders stability to eRNA as these lack loose ends. Further, eRNA is incapable of eliciting immune responses as they are composed of regular unmodified nucleotides. It thus allows greater therapeutic efficacy and enhanced expression of therapeutic proteins. Also, eRNA backbone is modifiable which makes it easy to insert or swap therapeutic genes (Benova et al., 2026). However, delivery methods for eRNA *in vivo* are still not devised Additionally, development of another circular RNA technology platform (oRNA) can enhance the therapeutic potential of circular RNA. Despite multiple advantages, circRNA applications are limited only to preclinical research due to factors as limited understanding of circRNA stability, lack of prospective studies, etc. Altogether, the migration and development of circRNA towards clinical testing represents the faster-evolving area.

**TABLE 5:** Representative circRNAs implicated in cancer and their reported functions.

circRNA	Cancer types	Expression pattern	Molecular mechanism	References
circACVR2A	Hepatocellular carcinoma	Upregulated	Promote the proliferation and migration of cells, leading to increased invasion of the cancer	(Alzahrani, 2026)
circPVT1	Triple negative breast cancer	Upregulated	Enhances tumourigenicity	(Zhang et al., 2026)
circIGF2BP3	Non-small-cell lung carcinoma	Upregulated	Leads to evasion of tumour from immune system	(Ou et al., 2025)
circTRPS1	Bladder cancer	Upregulated	Provides tumour microenvironment	(Saadh et al., 2025)
circRNA-BISC	Breast cancer	Upregulated	Overcome BETi resistance	(Guo et al., 2025)
circITGB6	Colorectal cancer	Upregulated	Enhances tumor metastasis	(Fang et al., 2025)
circ-hnRNPU	Gastric cancer	Downregulated	Suppress tumour development	(Dong et al., 2025)
circACTN4	Breast cancer	Upregulated	Promotes tumour invasion, and metastasis	(Abidin, 2025)
circDNMT1	Breast cancer	Upregulated	Activation of autophagy	(Singh et al., 2025)
circAMOTL1	Breast cancer	Upregulated	Promote tumour development	(Yang J. et al., 2025)
circSCRIB	Breast cancer	Upregulated stability of mRNA	Enhances migration, invasion and tumour formation	(Qiu et al., 2026)
circRMST	Neuroendocrine prostate cancer and small cell lung cancer	Upregulated	Promote tumorigenesis	(Li and Oser, 2025)
circACTN4	Intrahepatic cholangiocarcinoma	Upregulated	Promote tumour metastasis	(Louis and Coulouarn, 2022)
circIPO11	Hepatocellular carcinoma	Upregulated	promotion of tumour propagation	(Yousefnia, 2025)
circINSIG1	Colorectal cancer	Upregulated	Promote tumour proliferation and metastasis	(Margvelani et al., 2025)
circMRPS35	Hepatocellular carcinoma	Upregulated	Confers cisplatin resistance	(Li et al., 2022)
circASK1	Lung adenocarcinoma	Downregulated	Confers enhanced sensitivity to gefitinib	(Sun et al., 2026)
circHNRNPU	Multiple myeloma	Upregulated	Promote MM progression	(Tang et al., 2022)
circEIF6	Triple-negative breast cancer	Upregulated	Promote tumour proliferation and metastasis	(Nunes et al., 2025)

While ASOs, siRNAs, miRNAs, mRNAs, and circRNAs primarily exert their therapeutic effects through modulation of gene expression or protein production, another important class of RNA therapeutics functions through direct molecular recognition of target biomolecules. RNA aptamers act as highly specific binding ligands capable of modulating protein activity, thereby expanding the therapeutic applications of RNA beyond gene silencing and protein replacement strategies.

## 6 RNA aptamers as therapeutic agents

**FIGURE 8:**
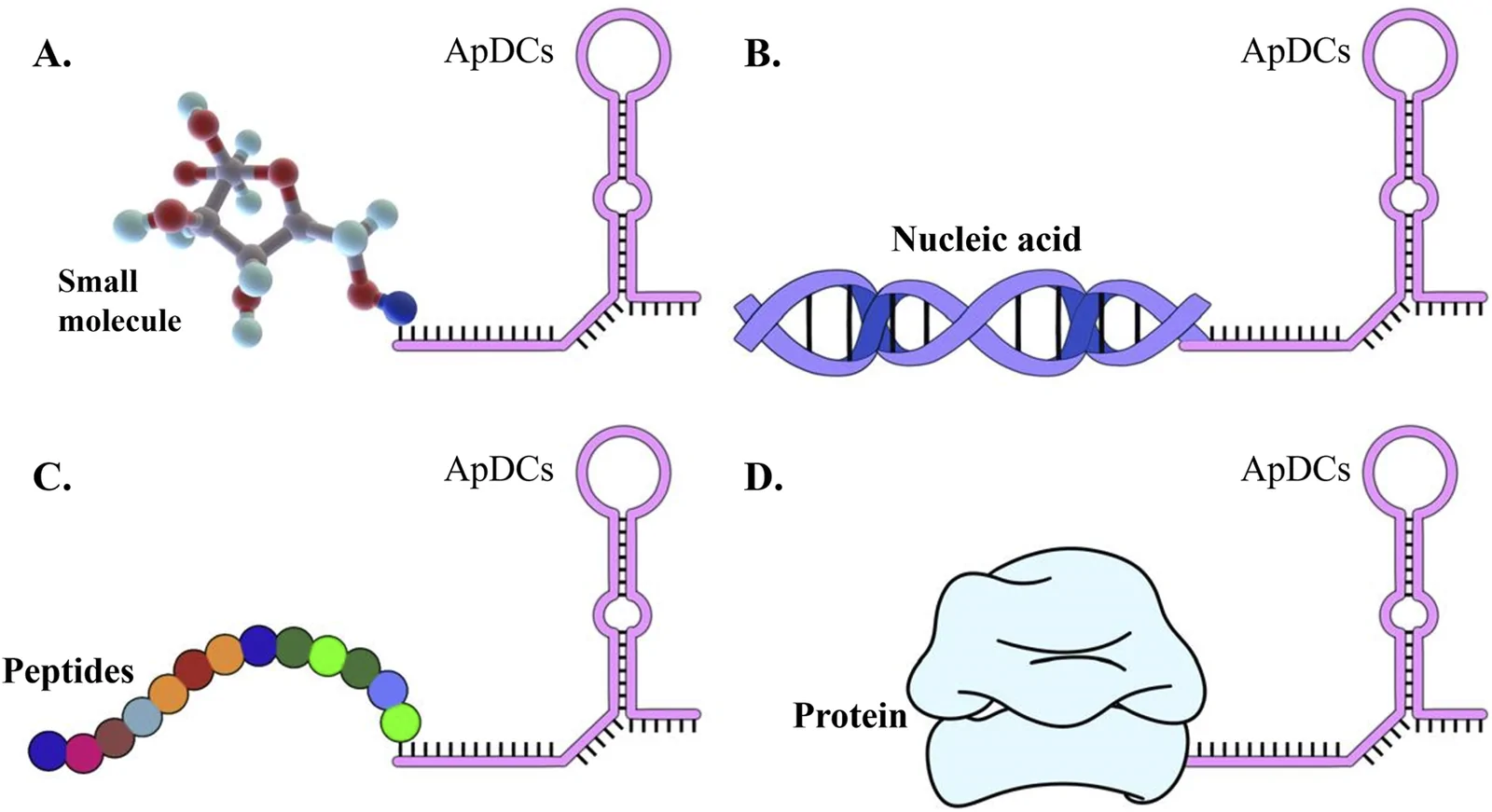
Depiction of mode of action of RNA aptamers, that are short single-stranded RNA molecules, which can interact with peptides, proteins, metal ions and other biomolecules with stronger affinity and specificity, hence suppressing their function (Zhu et al., 2022). Representative interaction of ssRNA with: **(A)** Small molecule, **(B)** Nucleic acid, **(C)** Peptides and **(D)** Proteins.

RNA aptamers are short, single-stranded nucleic acid molecules capable of folding into unique three-dimensional structures that bind target proteins, peptides, metal ions, and other biomolecules with high affinity and specificity. Through this selective molecular recognition, aptamers can modulate biological activity and serve as therapeutic agents in a variety of disease settings (Germer et al., 2013). The word aptamer comes from latin aptus, meaning fit and the Greek meros, means part or region. Aptamers are actually single-stranded, short RNA molecules that exhibit propensity to bind to proteins, peptides, metal ions and various biomolecules with high affinity and specificity leading to inhibition of their functions (Figure 8). The folded structure assumed by aptamers are thought to interact with the target moiety directly. Molecular level studies of pathogenesis of Human Immunodeficiency virus (HIV) and adenovirus demonstrated that viral-encoded small, structured RNA binds to endogenous proteins, which assists viral replication and leads to mitigation of antiviral activity by hosts (Takahashi et al., 2019; Mueller et al., 2025). Hence, these findings generated the idea that protein-binding nucleic acids can act as therapeutic agents.

**FIGURE 9:**
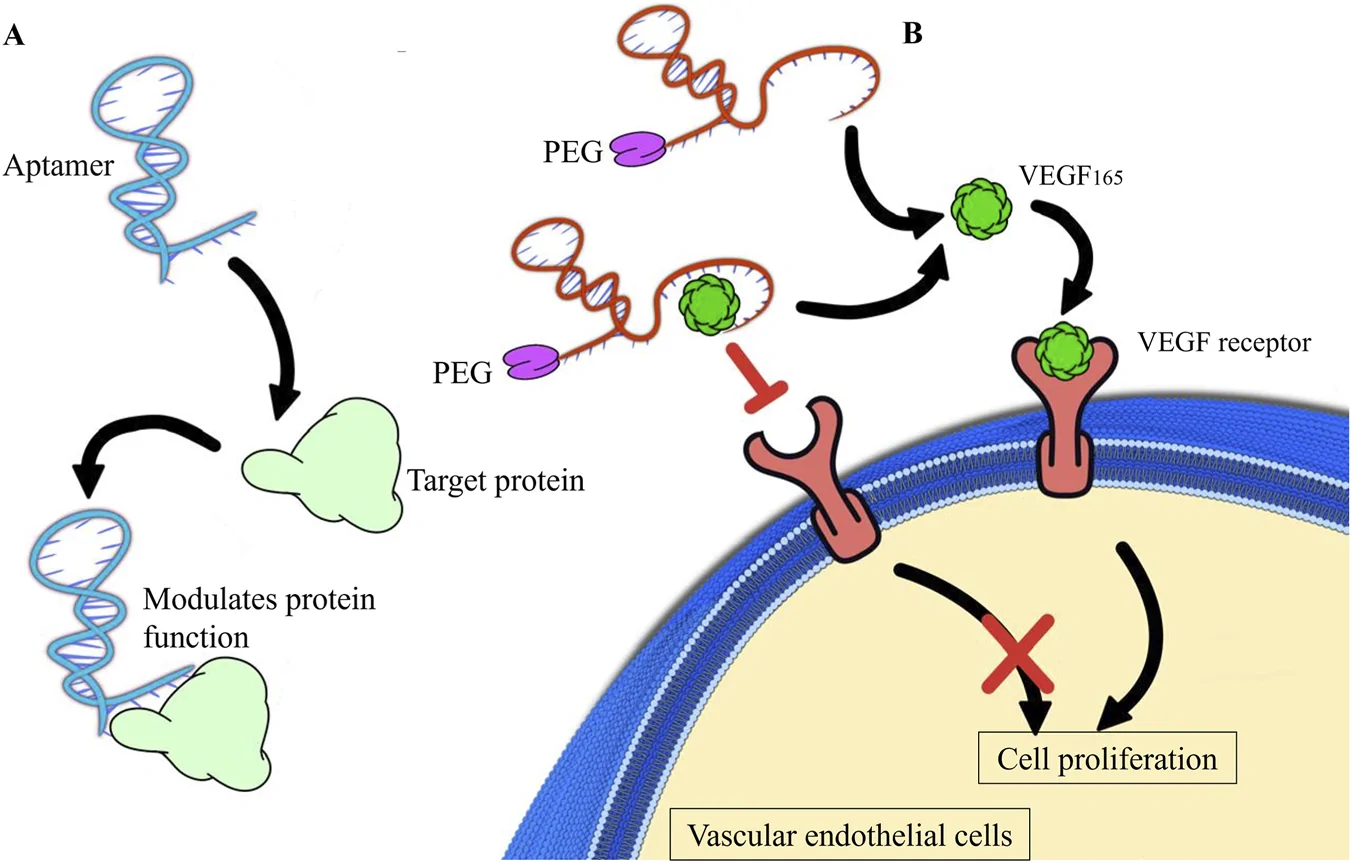
Illustration/Schematic diagram indicating mechanism of action of RNA aptamers. Aptamers exhibit propensity to attach to the crucial domains of target proteins, thus preventing their interaction with natural ligands. The recognition of target moiety occurs via complex shapes such as loops and hairpins and is governed by non-covalant interactions. Pegaptanib strongly interacts with Vascular Endothelial Growth factor (VEGF), thus blocking the interaction of its receptor, affecting angiogenesis (Roshan Mehr et al., 2024). **(A)** Mechanism of action for aptamer. **(B)** Pegaptanib.

In 1990, Sullenger et al. for the first time demonstrated binding of RNA aptamers to a viral protein that blocked the replication of virus (Thakur and Thakur, 2026). First therapeutic aptamer was developed nearly 25 years ago and till date only one aptamer got the approval for clinical use for the treatment of macular degeneration of eye, while others are in pre-clinical and clinical trial phase. Pegaptanib (Macugen) is the first FDA-approved aptamer drug that targets vascular endothelial growth factor (VEGF) for the treatment of age-related macular degeneration (Derham and Brown, 2024). It is comprised of 28-nucleotides with two Polyethylene glycol (PEG) moieties present at the end. It shows interaction with 165-isoform of VEGF, disrupting its interaction with its receptor protein, hence affecting downstream effects of VEGF signalling, leading to suppression of cell proliferation (Figure 9) (Bege et al., 2025). Another aptamer Avacincaptad pegol was approved for geographic atrophy (GA) secondary to AMD in 2023 (Shakeel et al., 2024). Other areas having promising potential for usage of RNA aptamer based drugs include thrombosis and oncology. The major reason for lagging behind of aptamer field was strong allergic reactions by the drug Pegnivacogin (an aptamer anticoagulant that causes inhibition of Factor IXa), leading to early termination of phase 3 clinical trials (Di Ruscio and De Franciscis, 2022). The hurdles in development of aptamers as major therapeutic agents involve concerns related to safety of these drugs, efficacy and delivery systems to be employed. Poor stability of RNA aptamers and rapid renal clearance act as hurdles for RNA to be used in therapeutics. Hence, certain chemical modifications can be incorporated during RNA aptamer development to enhance RNA stability and prevention of activation of immune responses. Development of various aptamers are going on for visual disorders, inflammation and oncology that are in pre-clinical or clinical trials. Given table enlists the aptamer-based drugs that are approved by FDA as well as the ones currently under the clinical trials (Table 6).

**TABLE 6:** Representative aptamer therapeutics in clinical development and approved clinical use (updated through 2026).

Name	Company/Brand	Molecular target	Application	Development status (2026)
Pegaptanib (Macugen)	Eyetech Pharmaceuticals/Pfizer	VEGF 165	Neovascular wet (age-related) vascular degeneration	Approved
Avacincaptad pegol (Izervay formerly Zimura)	Iveric Bio/Astellas Pharma	Complement C5	Geographic atrophy secondary to age-related macular degeneration	Approved
NOX-A12 (Olaptesed pegol)	NOXXON Pharma	CXCL12	Glioblastoma, Colorectal and pancreatic cancer, Chronic Lymphocytic Leukemia, Multiple Myeloma	Phase II
ApTOLL	AptaTargets S.L.	TLR4	Acute ischemic stroke	Phase Ib/IIa
BC007	Berlin Cures GmbH	Functional autoantibodies against GPCRs	Long COVID-19, dilated Cardiomyopathy	Phase II
BT200	Medical University of Vienna	von Willebrand factor (vWF)	Von Willebrand Diseases, Hemophilia A	Phase II
AON-D21	Aptarion Biotech AG	Host inflammatory mediators	Community-acquired pneumonia	Phase II
BB-031	Basking Biosciences	von Willebrand factor (vWF	Acute ischaemic stroke	Phase II
NOX-H94 (Lexaptepid pegol)	NOXXON Pharma	Hepcidin	Anemia of Chronic Diseases	Phase II
Apta-1	Aptahem AB	Coagulation and inflammatory pathways	Sepsis, Inflammation	Phase I
AS1411	Antisoma Research	Nucleolin	Acute Myeloid Leukemia and advanced solid tumors	Phase II

The relatively limited number of approved aptamer therapeutics highlights the challenges associated with translating aptamer technology into clinical practice. Aptamers possess high target specificity and affinity comparable to monoclonal antibodies; however, rapid renal clearance, susceptibility to nuclease degradation and pharmacokinetic limitations have hindered broader clinical adoption. Pegaptanib and avacincaptad pegol achieved regulatory approval largely because they are administered through intravitreal injection, which allows localized target engagement while minimizing systemic exposure. Unfortunately, several systemic aptamer candidates failed to progress due to safety concerns or insufficient efficacy. One notable example is pegnivacogin, an anticoagulant aptamer targeting factor IXa, whose phase III clinical development was terminated following severe hypersensitivity reactions. These findings emphasize the importance of delivery route, molecular stability and safety optimization in determining the clinical success of aptamer therapeutics (Akhtar F. et al., 2026).

Aptamers are known as chemical antibodies as they are synthetic in nature and exhibit specificity and action in a similar way as antibodies. They are also known as nucleic-acid monoclonal antibodies (mAbs). Aptamers have tendency to assemble into three-dimentional configrations that are capable of forming aptamer-target complexes same as observed between antigen and antibodies. They have been shown to bind to their targets with high affinity in a similar way as antibodies exhibiting dissociation constants (Kd), ranging from nanomolars to picomolars (Domsicova et al., 2024). Aptamers show high specificity as well as high affinity binding towards the target molecules. Some of the major differences have been outlined between antibodies and aptamers below (Table 7).

**TABLE 7:** Key differences between aptamers and antibodies.

Aptamers	Antibodies
Entire selection is a chemical process carried out *in vitro* and can therefore target any protein	Selection requires a biological system, therefore difficult to raise antibodies to toxins (not tolerated by animal) or non-immunogenic targets
Can select for ligands under a variety of conditions for *in vitro* diagnostics	Limited to physiological conditions for optimizing antibodies for diagnostics
Iterative rounds against known target limit screening processes	Screening monoclonal antibodies is time-consuming and expensive
Uniform activity regardless of batch synthesis	Activity of antibodies varies from batch to batch
Pharmacokinetic parameters can be changed on demand	Difficult to modify pharmacokinetic parameters
Investigator determines target site of protein	Immune system determines target site of protein
Wide variety of chemical modifications to molecule for diverse functions	Limited modifications to molecule
Unlimited shelf life	Limited shelf life
No evidence of immunogenicity	Significant immunogenicity
Cross-reactive compounds can be isolated using toggle strategy to facilitate preclinical studies	No method for isolating cross-reactive compound
Aptamer-specific antidote can be developed to reverse the inhibitory activity of the drug	No rational method to reverse molecules

Aptamer selection is done by the method of Systemic Evolution of Ligands through Exponential Enrichment (SELEX) (Kohlberger and Gadermaier, 2022). The pool of RNA molecules is tested for interaction with the target protein, followed by reverse transcription and amplification of selected molecules that can bind efficiently to finally get enriched pool (Didarian et al., 2025). This enabled the isolation of aptamers for different kinds of targets. With the aim of improving selection efficiency, advancements have been made for designing of initial library, identification and preparation of targets followed by PCR amplification. Magnetic-assisted rapid aptamer selection (MARAS) technique is a recently developed technique for the same (Cesarini et al., 2025). Further manipulation of structures of aptamers impact their pharmacological properties that can be tailored to achieve desired effects for clinical needs.

Aptamers show preferential interaction with positively charged surface of target proteins as the backbone linkages are negatively charged. However, crystal structures show that the affinity between aptamer and target is mainly mediated by electrostatic forces. In order to have high conformational heterogeneity and greater target specificity, several additions can be done in the structure of aptamers. Firstly, chemical modifications can be incorporated on sugar/phosphodiester backbone to provide protection to aptamers against endogenous nucleases (Cheng et al., 2025). Aptamers having L-ribose-based nucleotide instead of natural D-nucleotides are developed by NOXXON Pharma. These are nuclease-resistant aptamers known as Spiegelmer (Amiri Hezave et al., 2026). Few Spiegelmers such as NOX-E36, NOX-H94 and NOX-A12 have entered the clinical trials (Elmanzalawy, 2026). Phosphorothioate and boranophosphate bonds are used in place of natural phosphodiester linkage (Akhtar U. et al., 2026). Secondly, reduced length of aptamer would lower the cost of drug manufacturing and will reduce unpredicted toxicity. Other modifications involve addition of carrier molecules such as polyethylene glycol (PEG) or cholesterol at ends of aptamers for enhancement of t1/2 to several hours (Zhou et al., 2024).

Aptamers are mainly designed for targeting intracellular, extracellular and circulating proteins. They can increase or decrease the interaction of target proteins with other protein molecules, can alter the activity and localization of proteins and can also help in drug delivery. Aptamers exhibit broad range of target specificity, better thermal stability and synthetic feasibility. All these factors pave way for RNA aptamer therapeutics. With progress and advancements in research, aptamers could be anticipated to play an indispensable role in therapeutics for a wide range of diseases.

Further, development of aptamer-drug conjugates (ApDCs) have exploited the excellent targeting ability of aptamers in therapeutic field (He et al., 2023). It involves covalent or non-covalent attachment of therapeutic payload with a defined aptamer to specifically deliver the drug at a particular target site and concentrating the drug there. This mechanism of ApDCs involves strong binding to surface followed by receptor-mediated endocytosis. Upon internalization, linker is cleaved in the endo-lysosomal microenvironment to release the therapeutic payload. ApDC platform exhibit remarkable flexibility as it can carry diverse range of payloads from small molecules like GEM, MMAE to sophisticated molecules like proteolysis-targetting chimeras (PROTACS) (Zhou et al., 2026). Advancements in ApDC designing can address challenges as acquired drug resistance and selective removal of cancer stem cells. Many combination strategies can lead to development of “all-in-one” ApDCs carrying multiple therapeutic moieties. Hence, ApDCs represent highly programmable and modular drug delivery platform. Cancer-targeted therapy employ ApDCs carrying small molecule drugs. For instance, ApDCs targeting PTK7 Receptor in haematological malignancies were developed by Sicco et al. Several recent studies and pre-clinical trials have shown promising insights into aptamer-based targeting.

Despite the diversity of RNA therapeutic modalities described above, their successful clinical translation ultimately depends on efficient delivery to target tissues and cells. Delivery challenges, including molecular instability, rapid clearance, limited cellular uptake, and off-target distribution, remain common obstacles across RNA-based therapies. Consequently, the development of safe and effective delivery platforms has become a central focus in the advancement of RNA medicine (Bathula et al., 2024). The following section discusses the major delivery strategies that have enabled the clinical success of RNA therapeutics.

## 7 Delivery systems of RNA therapeutics

Delivery technologies are critical determinants of the therapeutic efficacy and clinical applicability of RNA-based medicines. Regardless of their mechanism of action, RNA molecules require protection from degradation and efficient transport to target cells to achieve their intended biological effects. RNA-based therapeutics present tremendous opportunities for precise intervention at the level of gene regulation or protein synthesis, making them suitable candidates for the treatment of disorders with well-characterized genetic underpinnings. Successfully modulating gene expression, however, requires overcoming a myriad of physiological and cellular barriers that fluctuate with tissue type, environmental influences, and developmental state (Zhu et al., 2022; Luo and Chen, 2024). The targeted delivery of RNA drugs is thus a major challenge, involving sequential processes such as extravasation from the vasculature, cellular uptake, and if the RNA is encapsulated effective release within the intracellular space (Curreri et al., 2023). Once inside the body, nucleic acid therapeutics are prone to degradation via filtration organs like the kidneys, and may be eliminated before reaching their intended site of action. Circulating RNA molecules must also evade inactivation by immune surveillance mechanisms and phagocytic cells before exerting biological effects. To address these hurdles, innovative delivery platforms have been developed including LNPs, polymer-based carriers, viral vectors, and chemically modified conjugates which enhance tissue specificity, stability, and cellular entry while minimizing toxicity and off-target clearance (Zhang et al., 2023; Pozdniakova et al., 2025). Continued refinement of these delivery systems is critical to enable the therapeutic potential of RNA medicines across diverse clinical contexts (Paunovska et al., 2022).

Achieving tissue-specific delivery remains a major challenge in the development of RNA therapeutics. While the liver has emerged as the most accessible target organ owing to its physiological role in nanoparticle uptake and the availability of receptor-targeting approaches, efficient delivery to other tissues such as the brain, lungs, skeletal muscle, and solid tumors remains considerably more (Dweh et al., 2025). Development of cell-specific ligands, biomimetic nanoparticles, and receptor-mediated targeting strategies is therefore an active area of investigation aimed at expanding the therapeutic reach of RNA-based medicines.

To overcome the inherent challenges associated with RNA delivery, researchers have devised a variety of viral and non-viral delivery platforms aimed at protecting RNA molecules, improving the precision of delivery to target cells, and limiting off-target effects (Paunovska et al., 2022). Adeno-associated viruses have been employed for viral-delivery methods while nanoparticle based delivery act as a major non-viral RNA delivery vector. Although viral vectors have historically been utilized for nucleic acid delivery, this overview concentrates on the significant progress made with non-viral carriers. Among these, nanoparticles have emerged as a leading class of delivery vehicles, owing to their capacity to physically shield RNA cargo from enzymatic degradation. Furthermore, depending on their chemical composition and surface properties, nanoparticles can facilitate cellular internalization and promote escape from endosomal compartments (Shin et al., 2018). Polymers are frequently harnessed in nanoparticle formulation due to their broad chemical diversity and tunable characteristics, making them invaluable in engineering efficacious RNA delivery systems (Huang et al., 2022).

### 7.1 Lipid-based nanoparticles

Due to the ionic charge and considerable size of RNA therapeutics, designing effective delivery systems is crucial for facilitating their passage across cellular membranes. Although nucleotide chemical modifications improve resistance against nucleases and reduce immune system activation, significant challenges remain to ensure stability, transport kinetics, tissue-specific targeting, and overall safety and efficacy of RNA drugs. Among various delivery platforms, lipid-based nanoparticles (LNPs) have emerged as the most clinically successful non-viral delivery technology for RNA therapeutics. Their pivotal role in enabling the delivery of mRNA COVID-19 vaccines and several approved siRNA-based medicines has established LNPs as a cornerstone of modern RNA drug development (Saini et al., 2026).

These systems offer advantages including straightforward formulation processes, excellent biocompatibility, and the capability to carry substantial therapeutic payloads (Buse and El-Aneed, 2010). LNPs, composed of biocompatible and biodegradable lipid vesicles, have gained significant attention as highly effective non-viral carriers for delivering nucleic acids. The lipid bilayer encasing the nucleic acid payload not only shields RNA from degradation but also plays a critical role in ensuring delivery to target cells with optimal stability and functionality. The lipid composition of this bilayer greatly influences the performance of LNPs. Typically, these nanoparticles consist of four main components: structural lipids, cholesterol, ionizable cationic lipids, and stealth lipids. Structural lipids, which are predominantly neutral phospholipids, provide the fundamental scaffold that maintains nanoparticle integrity (Xu et al., 2025). This lipid architecture is finely tuned to balance protection, cellular uptake, and endosomal escape, enhancing the overall therapeutic efficiency of RNA delivery systems. LNPs offer enhanced safety and low immunogenicity than viral vectors. LNPs have revolutionized RNA therapeutics field by minimizing the challenges associated with safety and delivery efficiency. The approved Pfizer-BioNTech’s Comirnaty and Moderna’s Spikevax has utilized LNP-mRNA as efficient vaccine platform (Kim et al., 2026).

Despite the remarkable success of LNPs technologies, endosomal escape remains one of the major barriers to efficient intracellular RNA delivery (Pei, 2025). Following cellular uptake, a large proportion of internalized nanoparticles become trapped within endosomal compartments and are subsequently degraded before releasing their RNA cargo into the cytoplasm. Consequently, only a small fraction of delivered RNA reaches its intracellular target site. To address this limitation, considerable efforts are focused on the development of ionizable lipids, pH-responsive materials, and membrane-disruptive formulations capable of enhancing endosomal release and improving therapeutic efficacy (Chahal et al., 2026).

Cationic lipids in LNPs play a crucial role by facilitating electrostatic interactions with the negatively charged nucleic acid molecules, thereby enhancing both encapsulation efficiency and transfection performance (Sun and Lu, 2023). These lipids form complexes known as “lipoplexes” through electrostatic binding with nucleic acids, a mechanism widely utilized in laboratory settings for nucleic acid transfection applications, including techniques employing reagents like Lipofectamine RNAiMAX (Cocchiararo et al., 2022). Phospholipids, characterized by polar head groups and hydrophobic tails, self-assemble in aqueous environments into liposomes spherical vesicles constructed from one or more phospholipid bilayers encapsulating an aqueous core. These versatile vesicles serve as customizable delivery vehicles capable of carrying a wide range of therapeutic agents (van der Koog et al., 2022; Zinger et al., 2020). The structural organization of liposomes allows modulation of their surfaces to optimize delivery characteristics and target specificity (Tenchov et al., 2021).

Liposomes offer the advantage of structurally resembling cell membranes, which allows them to effectively encapsulate mRNA when combined with cationic lipids. The assembly of RNA-liposome complexes relies on the electrostatic attraction between the negatively charged mRNA molecules and the positively charged lipid components. Incorporation of cholesterol-modified lipids into these complexes further stabilizes the structure and enhances transfection efficiency (Belhadj et al., 2023). Additionally, polyethylene glycol (PEG)-conjugated lipids are incorporated into LNPs to finely control particle size, reduce aggregation tendencies, and extend systemic circulation time. The PEG coating forms a steric barrier that minimizes protein adsorption, especially of opsonins, thereby decreasing the likelihood of recognition and clearance by the immune system (Yavuz, 2022).

The efficiency of LNPs delivery is significantly influenced by their acid dissociation constant (pKa), which dictates the ionization state and surface charge of the nanoparticles. Positively charged LNPs protect negatively charged RNA molecules from enzymatic degradation and facilitate cellular internalization via receptor-mediated endocytosis. As the endosome matures and acidifies, lowering its pH from approximately 7 to 5.5, LNPs with an optimal pKa become protonated, which results in a buffering effect that disrupts the endosomal membrane. This disruption causes osmotic swelling and eventual rupture, enabling the therapeutic siRNA to escape into the cytoplasm. Once released, siRNA dissociates from the nanoparticles and associates with the RNA-induced silencing complex (RISC), which guides the sequence-specific cleavage of target RNA molecules (Iwakawa and Tomari, 2022). The precise tuning of nanoparticle pKa is thus crucial for maximizing endosomal escape efficacy and therapeutic outcomes.

Despite advancements, vesicles carrying RNA therapeutics remain vulnerable to degradation within endosomal compartments. To enhance the release of RNA cargo, synthetic cationic lipids such as 1,2-dioleoyl-3-trimethylammonium-propane (DOTAP) are incorporated into LNPs. Within the cellular environment, endogenous anionic lipids interact with these synthetic cationic lipids, leading to neutralization that facilitates the liberation of the RNA payload (Miatmoko et al., 2023). Achieving optimal encapsulation and transfection efficiency in LNP formulations necessitates a delicate optimization of various parameters, including lipid composition, surface charge, particle size, and surface modifications.

### 7.2 GalNAc-conjugated delivery systems

N-acetylgalactosamine (GalNAc) conjugation has emerged as one of the most successful targeted delivery approaches for RNA therapeutics, particularly for liver-associated diseases. GalNAc ligands bind with high affinity to the asialoglycoprotein receptor (ASGPR), which is abundantly expressed on hepatocytes (O'Sullivan et al., 2022). Following receptor-mediated endocytosis, GalNAc-conjugated RNA molecules are efficiently internalized into liver cells, enabling potent gene silencing while minimizing systemic exposure. Several clinically approved siRNA therapeutics employ this strategy owing to its high tissue specificity, favorable safety profile, and reduced dosing requirements (Tompach et al., 2026). However, the applicability of GalNAc-based delivery remains largely restricted to hepatic targets, highlighting the need for analogous targeting systems for extrahepatic tissues.

### 7.3 Polymer nanomaterials

Polymer nanoparticles constructed from PLGA offer numerous clinical advantages, such as biodegradability, biocompatibility, and controlled drug release over extended periods. Despite these benefits, the encapsulation and delivery efficiency for small RNA molecules like siRNA remain limited due to PLGA’s neutral charge at physiological pH, which hinders effective interaction with the negatively charged RNA backbone. Early studies have shown that PLGA nanoparticles alone yield low siRNA encapsulation efficiency and insufficient intracellular release, resulting in suboptimal gene silencing outcomes. To enhance performance, researchers have incorporated cationic polymers such as polyethylenimine (PEI) into the PLGA matrix, significantly improving siRNA loading, cellular uptake, and sustained release profiles. The incorporation of PEI promotes nanoparticle interactions with the RNA cargo, facilitating better protection and delivery to the cytoplasm. While PEI-enhanced PLGA nanoparticles display improved gene silencing efficacy, concerns remain regarding the cytotoxicity of some polycationic agents, prompting exploration of safer alternatives such as Poly-L-lysine. Overall, modifying PLGA nanoparticles with cationic polymers offers a promising platform for RNA-based therapeutics, balancing safety, stability, and delivery efficiency (Friesen and Blakney, 2024).

A variety of amine-containing polymers, including polyethylenimine (PEI) and poly (L-lysine) (PLL), have been chemically modified to facilitate targeted delivery of mRNAs to specific cell types. Recent advancements have explored lipid-polymer hybrid nanoparticles as a means to enhance stability in systemic circulation and improve delivery efficiency (Soomherun et al., 2024). Although polymers such as PEI, PLL, and poly (amidoamine) (PAMAM) dendrimers demonstrate high efficacy in RNA delivery, their potential cytotoxicity poses significant limitations. To address these concerns, polyethylene glycol (PEG) has been integrated into these polymeric systems to reduce toxicity without compromising therapeutic performance. Beyond synthetic polymers, natural polysaccharides like dextran and chitosan have also emerged as promising carriers for mRNA delivery due to their biocompatibility and biodegradability (Petrovici et al., 2023; Kowalski et al., 2019).

Synthetic cationic polymers, which can be functionalized with various targeting ligands, have been widely employed in the formulation of RNA-based nanoparticle therapeutics (Friesen and Blakney, 2024). Polyethylenimine (PEI), a non-biodegradable polymer, is one of the most extensively used carriers for delivering siRNAs and self-amplifying mRNAs due to its strong ability to complex nucleic acids and facilitate cellular uptake (Thomas et al., 2019). Similar to liposomal delivery systems, these polymers utilize diverse intracellular drug release mechanisms. A common pathway involves the cleavage of disulfide bonds within the polymer matrix, triggered by the reductive environment of the cytoplasm, which enables controlled release of the RNA payload. Ongoing research aims to optimize polymer properties to balance transfection efficiency with biocompatibility and degradability.

Dendrimers, a unique class of polymers, possess distinctive features that make them highly advantageous for RNA therapeutic delivery. Their well-defined branched architecture provides chemical versatility, stability, and polyvalency, enabling multiple surface modifications for targeted delivery. These polymers exhibit low cytotoxicity, high solubility, self-assembly capabilities, and excellent biocompatibility. Notably, dendrimers can be engineered to incorporate various ligands on their surfaces, enhancing specificity toward particular cell types. For instance, dendrimer-based nanoparticles have been successfully utilized for targeted *in vivo* delivery of siRNA and saRNA to silence Hsp27, demonstrating their potential in gene silencing applications (Dzmitruk et al., 2018). The multivalent nature and customizable surface chemistry of dendrimers contribute significantly to their efficacy as RNA carriers, positioning them as promising candidates in advanced RNA-based therapeutics.

The effectiveness of RNA delivery by polymer-based nanoparticles is largely influenced by their physicochemical properties, such as surface charge, biodegradability, and molecular weight. One successful approach to enhance RNA delivery involves incorporating cationic functional groups, exemplified by polymers like chitosan, which facilitate strong interactions with negatively charged RNA molecules. Polymer nanoparticles play an essential role as versatile and adaptable non-viral vectors in RNA therapeutics, allowing customization to address diverse therapeutic challenges.

### 7.4 Silica nanoparticles

Mesoporous silica nanoparticles (MSNs) have emerged as a highly promising platform for RNA therapeutic delivery by addressing key challenges related to RNA stability and efficient cellular uptake. These nanoparticles provide a versatile system capable of co-delivering RNA molecules alongside small-molecule drugs, enabling synergistic combination therapies particularly effective in oncology and other medical fields (MacLeod and Crooke, 2017; Kwon et al., 2013). The uniquely large surface area and extensive pore volume inherent to MSNs allow for substantial RNA loading capacity (Castillo et al., 2017). Moreover, the adjustable pore size along with modifiable surface chemistry facilitate controlled, stimulus-responsive release and targeted delivery of therapeutic agents (Möller et al., 2016; Liu and Xu, 2019). Functionalizing MSNs with polymers, proteins, or stimuli-sensitive moieties enhances their ability to respond to intracellular cues such as changes in pH, ATP levels, or enzyme concentrations, triggering precise cargo release at the desired site of action (Buchman et al., 2013; Song et al., 2017). Together, these attributes highlight MSNs as a versatile and effective vector for RNA delivery applications. Among the many potential uses of MSN-mediated RNA delivery, cancer treatment especially the ability to address multidrug resistance (MDR) stands out as highly promising. The use of MSNs for co-delivering siRNA with chemotherapeutic drugs has shown promise against MDR, as it allows simultaneous inhibition of resistance pathways and induction of cytotoxicity (Hanafi-Bojd et al., 2016). MSNs have also been explored as carriers for other nucleic acids, including DNA and mRNA, thereby extending their potential applications in biotechnology. Such applications span from live-cell and biosensing platforms to gene transfection and silencing (Li et al., 2011; Paris and Vallet-Regí, 2020). MSNs offer a flexible and novel platform for delivering RNA therapeutics, with uses that extend to cancer therapy, gene therapy, and broader biomedical applications. The capacity of MSNs to co-deliver RNA with additional therapeutic agents, along with their tunable features, opens avenues for designing more precise and advanced treatment strategies. Despite their promise, additional studies are needed to overcome challenges in clinical translation and to enhance the safety and effectiveness of MSN-mediated RNA delivery.

## 8 Clinical applications of RNA therapeutics in cancer

Cancer is primarily a genetic disorder, and its characteristic changes are frequently mirrored at the proteomic level, driving cell survival, unchecked growth, angiogenesis, and metastasis (Wacheck and Zangemeister-Wittke, 2006). Targeted therapies have revolutionized cancer treatment by allowing specific intervention against genetic alterations that drive the progress of tumor formation (Quemener et al., 2020). RNA-based therapeutics are emerging rapidly in oncology, offering solutions to limitations associated with traditional chemotherapy (Magoola and Niazi, 2025). Traditional radiation-based treatments and chemotherapeutic treatments harm and impair the activity of healthy cells. Hence, development of RNA therapies for treatment of cancer is quite promising since it selectively targets cancer cells without affecting healthy cells (Han et al., 2023). A significant advantage of RNA-based therapeutics is their ability to selectively target long non-coding RNAs (lncRNAs), which play crucial roles in regulating cancer-related molecular pathways in a cell type–specific manner. This targeted approach enhances therapeutic efficacy while simultaneously posing challenges in the course of clinical development (Coan et al., 2024). The development of RNA-based cancer treatments has been strongly linked to improvements in delivery technologies. In particular, nanoparticles most notably LNPs have shown great potential as versatile vehicles for transporting RNA therapeutics directly to tumor tissues. These nanocarriers are designed to overcome typical challenges of siRNA therapy, including poor delivery efficiency and unintended off-target effects (Miele et al., 2012). Earlier studies have shown LNPs encapsulating IL-12 mRNA to be used as therapeutic agents for hepatocellular carcinomas (HCC) (Wang et al., 2026). Furthermore, targeted delivery approaches, including pulmonary and intratumoral administration, have shown promising results in preclinical studies using murine models.

The high precision of ASOs in recognizing their target mRNA renders them powerful therapeutic tools for treating human cancers. ASOs are capable of precisely regulating the expression of target genes and inhibiting cancer cell malignancy. Proteases and their receptors, telomerase, fusion genes, the Bcl protein family, and several protein kinases have been major targets of antisense cancer therapy and have been extensively investigated. The precise expression patterns of ASOs enable them to target cancer cells selectively, minimizing damage to healthy tissues (Rehman et al., 2024). ASOs primarily function by promoting apoptosis and increasing the sensitivity of cancer cells to the growth-inhibitory effects of anticancer drugs. Integrating ASOs with cytotoxic drugs offers notable benefits in cancer therapy. Studies have demonstrated a synergistic antitumor effect when antisense treatments are combined with conventional chemotherapy (Biroccio et al., 2003). ASOs can also act as inhibitors of miRNAs, small non-coding RNAs involved in gene regulation. By binding to miRNAs, ASOs prevent their interaction with target mRNAs, which can relieve miRNA-mediated suppression of tumor suppressor genes or increase the expression of oncogenes (Barresi et al., 2022). This dual role highlights the adaptability of ASOs as a potential therapeutic approach in cancer management.

RNA interference (RNAi) is a natural cellular mechanism in which RNA molecules inhibit gene expression or protein synthesis by binding to and silencing specific mRNA targets. RNAi suppresses the expression of oncogenes and other cancer-associated genes, decreasing the production of proteins that promote cancer cell growth, survival, and metastasis (John et al., 2025).

Consequently, targeting oncogene expression can slow tumor progression, increase apoptosis in cancer cells, and enhance their responsiveness to treatments such as chemotherapy and radiation therapy. This targeted action highlights RNAi’s potential as a precise strategy for cancer therapy.

The application of mRNA vaccine for cancer treatment is progressing at a fast pace. mRNA-based treatments employ synthetic mRNA to generate therapeutic proteins or trigger an immune response against cancer cells. The mRNA vaccines can deliver specific mRNA sequences upon encoding oncogenic antigens that evokes a targeted response against cancer cells by host immune system (Yaremenko et al., 2025). These vaccine development needs personalized approach due to heterogeneity of cancer antigens in different individuals. Hence, it is designing requires identification of genetic sequences of the tumour so that vaccine can precisely target tumour-specific neo-antigens that renders enhanced specificity and efficacy to these vaccines (Li M. et al., 2025). The therapeutic effects of mRNA in cancer involve multiple sequential steps. First, synthetic mRNA is engineered to encode proteins that can either directly attack cancer cells or stimulate the immune system to target them. Delivery systems, such as LNPs, protect the mRNA from enzymatic breakdown and facilitate its efficient uptake by the intended cells. After administration, the LNPs carrying the mRNA are taken up by cells via endocytosis, releasing the mRNA into the cytoplasm (Pardi et al., 2018). Within the cytoplasm, ribosomes translate the synthetic mRNA into therapeutic proteins. These proteins can function in various ways, such as acting as tumor antigens, cytokines, or other immune-modulating molecules (Sahin et al., 2014). In cancer immunotherapy, mRNA-encoded proteins may serve as tumor-associated antigens presented on the cell surface, which activate dendritic cells and, in turn, stimulate T cells to target and eliminate cancer cells expressing the same antigens (Kranz et al., 2016; Diken et al., 2011). Additionally, some mRNA-derived proteins may exert direct therapeutic effects, such as inducing apoptosis in cancer cells or interfering with oncogenic signaling pathways (Hou et al., 2021). This demonstrates the versatility and promise of mRNA-based therapies in oncology. Employing combination therapies wherein multiple cytokines are analysed and combined to generate augmented therapeutic effects. Upon combining IL-12, IL-27, GM-CSF in LNPs have shown to generate very high antitumor effect by induction of NK and CD8^+^ T-cells.

Altogether, different types of RNA molecules as siRNA, mRNA, miRNA and aptamers can affect growth, progression and metastatic activity of tumors, hence targeting cancer cells with high sensitivity and selectivity (Magoola and Niazi, 2025). Presently, the US National Library of Medicine’s online database of clinical studies (ClinicalTrials.gov) found 116 clinical trials for mRNA cancer vaccines for pancreatic cancers, esophageal and thyroid cancers, leukemia, melanoma, cervical, ovarian and others (Zaidi et al., 2025). Further, vaccine EVM14 is a tumor-associated antigen (TAA) vaccine candidate whose IND (Investigational New Drug) application was authorized by US-FDA in 2025 (Lavigne, 2025). It is an mRNA-lipid nanoparticle mRNA-based cancer vaccine for non-small cell lung cancer (NSCLC) and head and neck cancer. Currently, developments in clinical trials for RNA-based cancer vaccines have gained momentum (Table 8).

**TABLE 8:** RNA-based cancer vaccines undergoing clinical trials.

Vaccine	Neoplasm	Platform	Results/Effectiveness	Clinical trial stage	Reference
mRNA-4157	Melanoma	Personalized mRNA	44% reduction in recurrence risk; 3-year sustained benefit	Phase 3 ongoing	(Lu et al., 2025)
Pancreatic Vaccine	PDAC	Personalized mRNA	4-year immune persistence; reduced recurrence in responders	Phase 1 completed	(Hussain and Fareed, 2025)
UF Glioblastoma	Brain Cancer	Layered nanoparticle mRNA	48-h immune activation; 4x survival extension in dogs	Phase 1 expanding	(Ahmed and Alam, 2025)
BNT111	Melanoma	Fixed antigen mRNA	Improved response vs. historical controls	Phase 2 ongoing	(Gazouli et al., 2025)

## 9 Clinical applications of RNA therapeutics in infectious disease

Recent advancements in understanding RNA biology and development of RNA-based drugs for various disorders have led to its involvement in infectious diseases too. The rise of infectious diseases is largely driven by the aggressive invasion of pathogens, such as microorganisms, into human and other host systems. The pathogens that causes infectious diseases exhibit rapid mutations that renders available methods ineffective as antigenic determinants tend to show continuous and rapid variations. RNA-based therapeutics represent one of the most ground-breaking developments in drug and vaccine innovation for combating these diseases. Traditional therapies have played major role in treatment of infectious diseases caused by different pathogens. However, the emergence of RNA therapeutics provides precise and targeted alternatives against the infectious agents (Pandey et al., 2021). The successful implication of RNA therapies for treatment of infectious diseases can employ involvement of different strategies that can affect the expression of disease-linked genes by replacement of mutated or abnormal genes or by lowering the expression of harmful proteins associated with the disease (Abdelzaher et al., 2021). Among them, ASOs have emerged as a promising strategy to fight infectious diseases, particularly in the face of escalating drug resistance. Their therapeutic potential is especially noteworthy for global health threats like tuberculosis. Although ASOs have been widely studied in cancer, neurodegenerative, and genetic disorders, their application in infectious diseases is still in its early stages. Even so, this field is advancing rapidly, with increasing research directed toward ASOs as a viable treatment option. The chemical adaptability of ASOs allows for modifications that minimize the side effects and improve delivery efficiency, strengthening their role as an advanced therapeutic platform (Buthelezi et al., 2023). Their high target specificity, limited influence on human gene expression, and straightforward design further enhance their appeal for targeting a broad spectrum of pathogens. However, achieving effective delivery of ASOs to intended sites remains a major hurdle. Overcoming this will require continued efforts to refine the delivery technologies, including the use of cell-penetrating peptides, LNPs, and DNA nanostructures (Anwar et al., 2023).

ASOs demonstrate remarkable versatility, with applications against a broad spectrum of pathogens, including both bacteria and viruses, making them a flexible tool in tackling infectious diseases. Overall, ASOs represent a promising strategy for managing infectious diseases, especially in the face of rising drug resistance, underscoring the urgent need for novel and effective treatments. Antiviral oligonucleotides, in particular, have been widely explored in preclinical studies as a means to inhibit virus-specific mRNAs. For instance, peptide-conjugated phosphorodiamidate morpholino oligomer (PPMO) ASOs have shown the ability to suppress Dengue virus replication by disrupting RNA translation and synthesis (Victorio et al., 2020; Holden et al., 2006; Phumesin et al., 2019).

Antisense technology has long been utilized to combat viral infections, with applications against both RNA and DNA viruses. Research has explored ASO-based therapies for RNA viruses such as influenza, dengue, coronavirus, and West Nile virus, as well as for DNA viruses like cytomegalovirus and hepatitis C virus (Table 8). RNA viruses are particularly suitable for ASO intervention since both their mRNA and genomic RNA can act as targets. This ability to simultaneously target multiple viral components provides the potential for complete viral clearance, making it a highly promising therapeutic strategy (Spurgers et al., 2008) (Table 9).

**TABLE 9:** Overview of antiviral oligonucleotides targeting viral pathogens.

Virus	ASO	Target	Mechanism	Reference
Dengue Virus	3′ stem-loop (3′SLT) PPMO	3′-SLT	Inhibits viral RNA translation	(Buthelezi et al., 2023)
	Vivo-MO-1	3′-SLT on 3′ UTR of genome		(Kim et al., 2026)
Influenza	P7-PMO	3′-terminal region of nucleoprotein viral genome RNA (NP-v3′)	Inhibits encapsidation of the negative viral RNA strand	(Maziec et al., 2025)
Ebola	Arginine rich PPMO	VP24 mRNA	Restores Interferon gene expression and associated responses	(Ogidi et al., 2025; Swenson et al., 2009)
Hepatitis B virus (HBV)	GalNAc- LNA (SSO)	HBV transcript	Reduces viral replication and HBsAg levels	(Hong and Rajwanshi, 2025; Javanbakht et al., 2018)
Human Immunodeficiency virus (HIV)	2′-deoxy-2′-fluoroarabinonucleotide (FANA)-ASO	HIV-1 genome	Inhibits viral replication	(Gotfrid et al., 2025)
Respiratory Synctial virus (RSV)	AUG-2 PPMO	RSV-L mRNA	Inhibits viral RNA dependent RNA polymerase	(Lai et al., 2008; Maziec et al., 2025)

At present, no FDA-approved oligonucleotides with antibacterial properties are available on the market. However, ASOs have emerged as a promising approach for developing novel antibacterial treatments, especially against multidrug-resistant pathogens like *Staphylococcus aureus*. These synthetic, single-stranded DNA molecules are engineered to specifically bind and suppress bacterial gene expression, offering distinct benefits in terms of precision and flexibility compared to traditional antibiotics (MacNair et al., 2024).

Chimeric ASOs combining first- and second-generation chemical modifications have shown effectiveness in inhibiting the growth of *S. aureus* (Angrish and Khare, 2023). By precisely targeting essential bacterial genes, these ASOs interfere with vital cellular functions, utilizing this as a potent strategy against drug-resistant pathogens.

Antibacterial ASOs provide an advanced strategy for combating multidrug-resistant bacterial infections, delivering exceptional specificity and the possibility of fewer side effects compared to conventional therapies (Saonere, 2011). As this field continues to progress, overcoming delivery challenges and refining chemical modifications will be essential to enhance the stability, efficacy, and overall therapeutic potential of ASOs as antibacterial agents.

RNA interference (RNAi) has emerged as a ground-breaking therapeutic strategy for infectious diseases, providing a precise means of silencing genes critical for pathogen replication and host–pathogen interactions (Dykxhoorn and Lieberman, 2005). This gene-silencing process utilizes siRNAs to promote the degradation of complementary mRNA, thereby suppressing gene expression (Zhou and Rossi, 2011). The ability to selectively silence virtually any gene makes siRNAs highly promising candidates for the development of treatments that targets a broad spectrum of human diseases, including infectious conditions (Sindhu et al., 2012). The regulation of pathogen invasion, replication and silencing of critical pathogen genes by employing siRNA, shRNA and miRNA is developed to combat infectious diseases.

In the field of viral infections, RNAi-based strategies have gained considerable interest, with several therapeutic candidates advancing to clinical trials (Tan and Yin, 2004). The swift progress of siRNA therapies for HIV (human immunodeficiency virus) highlights the strong potential of this approach. Extensive studies have demonstrated that RNAi can inhibit HIV-1 replication by targeting multiple viral and host genes (Jacque et al., 2002). These findings have paved the way for applying RNAi to other viral pathogens, including those responsible for respiratory diseases (Asha et al., 2018; Haasnoot et al., 2007).

Beyond viruses, RNAi therapeutics are also being explored for infectious diseases caused by bacteria, fungi, and parasites. By silencing essential genes within the pathogen or host factors required for their survival and replication, RNAi offers a versatile platform for designing novel antimicrobial strategies. The ability to rapidly develop siRNAs specific to pathogen sequences provides a powerful advantage in combating emerging infections and keeping pace with the rapid evolution of pathogens (Kreuze, 2014).

One of the major strengths of RNAi-based therapies is their ability to overcome the shortcomings of conventional drug treatments. Unlike traditional small-molecule inhibitors, siRNAs can be designed to silence virtually any gene, even those once considered “undruggable” (Alagia and Eritja, 2016). This flexibility opens the door to exploring novel therapeutic targets and creating more effective treatments for infectious diseases that have been difficult to control using standard methods.

Despite the strong potential of RNAi therapeutics, several challenges must be addressed before they can reach their full clinical application. One of the major hurdles is ensuring the efficient delivery of siRNA molecules to specific tissues and cells (Kanasty et al., 2013). Because siRNAs are relatively large and carry a negative charge, they struggle to cross cell membranes, making the development of effective delivery systems essential (Ahn et al., 2023).

Researchers have explored various strategies to overcome the challenges of siRNA delivery, including chemical modifications to improve siRNA stability and the development of advanced nanocarrier systems (Gangopadhyay and Gore, 2022; Gao et al., 2011). These strategies aim to enhance the pharmacokinetic behaviour of siRNAs, protect them from enzymatic degradation in biological environments, and facilitate efficient uptake by target cells (Isazadeh et al., 2023). Nanocarriers, such as LNPs, polymeric nanoparticles, and inorganic nanoparticles, have shown significant potential in improving both delivery efficiency and the therapeutic effectiveness of RNAi treatments (Chaturvedi et al., 2025; Ewe et al., 2017). In the context of respiratory infections, pulmonary administration of RNAi therapeutics has emerged as a highly promising approach (Bitko and Barik, 2007).

This non-invasive route enables direct delivery of therapeutics to the airways, which can potentially minimize systemic side effects. Nevertheless, the lungs present distinct anatomical, physiological, and immunological barriers that make effective drug delivery challenging. Addressing these obstacles is crucial for the successful development of inhalable RNAi-based therapies (Ren et al., 2025). Meanwhile, mRNA vaccines have emerged as a novel and promising platform for both preventing and treating infectious diseases, providing several advantages over conventional vaccine strategies.

These vaccines utilize mRNAs to instruct cells to produce specific antigens, enabling the immune system to recognize and combat pathogens efficiently. The flexibility in designing and modifying antigens, combined with the capacity for rapid scale-up to address emerging variants and the simplicity of large-scale production, underscores the substantial potential of mRNA vaccines in combating a wide range of infectious diseases (Al Fayez et al., 2023). The rapid development of mRNA vaccines has been supported by several key technological advances, particularly in the generation of high-quality, stable mRNA. These innovations have addressed major challenges, including improving stability, optimizing delivery systems, and enhancing immunogenicity, collectively paving the way for the successful clinical use of mRNA-based vaccines (Kutikuppala et al., 2024).

The effectiveness of mRNA vaccines has been clearly demonstrated in the fight against major infectious diseases, most notably through the rapid development and deployment of COVID-19 vaccines (Lamb, 2021). This remarkable achievement highlighted the capacity of mRNA technology to respond quickly and effectively to emerging infectious threats (Pardi and Krammer, 2024). Beyond COVID-19, mRNA vaccine platforms are being increasingly explored for other infectious diseases, including influenza, Zika virus, respiratory syncytial virus (RSV), HIV, and rabies. Several pharmaceutical companies, such as Moderna and Sanofi, are actively conducting clinical trials to develop mRNA-based vaccines for influenza and additional infectious diseases, emphasizing the broad applicability and promise of this innovative approach (De Clercq, 2006). Novel developments in the field of RNA therapeutics can revolutionize treatment of infectious diseases and can contribute towards global healthcare.

Collectively, advances in delivery technologies have significantly accelerated the clinical development of RNA-based medicines. However, each RNA therapeutic platform possesses unique advantages, limitations, and translational challenges that influence its suitability for specific clinical applications. A comparative evaluation of these modalities is therefore important for understanding their current status and future potential.

## 10 Comparative advantages and limitations of RNA therapeutic platforms

The rapid expansion of RNA-based therapeutics has generated multiple treatment modalities with distinct mechanisms of action, clinical applications, and developmental challenges. Although all RNA therapeutics aim to modulate disease-associated molecular pathways, their therapeutic success depends on factors such as target accessibility, delivery efficiency, durability of response, safety profile, and manufacturing feasibility (Table 10).

**TABLE 10:** Comparison of major RNA therapeutic modalities with respect to their mechanisms, advantages, limitations, clinical development status, and future therapeutic opportunities.

RNA platform	Principle mechanism	Key advantages	Major limitations	Current clinical status	Key future opportunities
ASOs	Modulation of RNA processing and gene expression	High target specificity; effective for genetic disorders	Repeated dosing, limited tissue penetration, delivery challenges	Multiple approved therapeutics	Expansion into neurological and personalized medicine applications through improved delivery technologies
siRNAs	RNA interference-mediated gene silencing	Potent and highly specific gene knockdown	Limited delivery beyond hepatic tissues	Multiple approved therapeutics	Development of extrahepatic targeting strategies and broader disease applications
miRNA therapeutics	Regulation of multiple genes and pathways	Ability to target complex disease networks	Off-target effects and pathway-wide perturbations	Clinical and preclinical development	Improved specificity and safer modulation of complex disease-associated gene networks
mRNA therapeutics	Transient expression of therapeutic proteins	Broad therapeutic versatility; vaccine and protein replacement applications	Instability, immunogenicity, storage and manufacturing challenges	Approved products and ongoing clinical trials	Cancer vaccines, protein replacement therapies, regenerative medicine, and personalized therapeutics
circRNA therapeutics	Sustained protein expression through enhanced molecular stability	Increased stability and prolonged activity	Early-stage development and limited clinical validation	Primarily preclinical	Long-duration protein expression, next-generation vaccines, and gene replacement strategies
RNA aptamers	Target-specific molecular recognition	High specificity, ease of synthesis and modification	Rapid clearance and delivery limitations	Limited approved therapeutics	Targeted drug delivery, diagnostic applications, and combination therapies

ASOs have demonstrated considerable clinical success in treating rare genetic and neurological disorders through selective modulation of RNA processing and gene expression (Rodriguez Carstens et al., 2026). Their high target specificity and relatively straightforward design make them attractive therapeutic agents. However, repeated administration, limited tissue penetration, and dependence on efficient delivery strategies remain important challenges. Similarly, siRNAs offer highly efficient and sequence-specific gene silencing through the RNA interference pathway and have achieved notable success in liver-targeted therapies (Iakovleva and de Jong, 2025). Nevertheless, extending their therapeutic efficacy to extrahepatic tissues remains a significant hurdle due to delivery-related constraints.

mRNA-based therapeutics differ fundamentally from gene-silencing approaches by enabling transient expression of therapeutic proteins (Deyhimfar et al., 2024). Their versatility has been demonstrated in vaccines, protein replacement therapies, and cancer immunotherapy (Żak and Zangi, 2025). Despite these advantages, challenges associated with molecular instability, storage requirements, manufacturing complexity, and innate immune activation continue to influence their broader clinical application. Emerging platforms such as circRNAs may overcome some of these limitations by providing enhanced stability and prolonged protein expression; however, their clinical development remains at an early stage and requires further validation.

RNA aptamers represent another promising class of RNA therapeutics that function through highly specific molecular recognition. Compared with conventional biologics, aptamers offer advantages including chemical synthesis, low batch-to-batch variability, and ease of modification. However, rapid renal clearance, limited *in vivo* stability, and delivery-related challenges have restricted their widespread clinical adoption. Likewise, miRNA-based therapeutics possess the unique ability to regulate multiple genes simultaneously, providing opportunities for treating complex diseases, although concerns regarding off-target effects and unintended pathway modulation remain significant.

Overall, the future success of RNA therapeutics will depend on balancing therapeutic efficacy with safety, stability, and delivery efficiency. Continued advances in RNA engineering, chemical modification, and targeted delivery technologies are expected to address many of the current limitations and further expand the clinical potential of RNA-based medicines.

Although substantial progress has been achieved across multiple RNA therapeutic platforms, several scientific and translational challenges continue to limit their broader clinical implementation. Understanding these challenges is essential for identifying future research priorities and guiding the next-generation of RNA-based medicines.

## 11 Mechanistic determinants of clinical success and failure of RNA therapeutics

The approved RNA therapeutics provides valuable insight into the molecular and pharmacological factors that determine clinical success. Although ASOs, siRNAs, mRNAs and aptamers utilize distinct mechanisms of action but successful therapeutics share several common characteristics.

Firstly, the approved RNA meds typically target genes with strong biological and genetic validation, ensuring that modulation of the target produces a predictable therapeutic outcome. For examples transthyretin (TTR), PCSK9, ALAS1, SOD1, APOC3 and SMN2. All of which have well-established roles in disease pathogenesis. Secondly the successful RNA therapeutics employ advanced delivery technologies that maximize target engagement while minimizing systemic toxicity. The widespread adoption of GalNAc conjugation and lipid nanoparticle systems has substantially improved intracellular delivery and pharmacokinetic performance (Wang et al., 2015; Ang et al., 2026). Lastly extensive chemical modifications have enhanced RNA stability, reduced nuclease degradation and limited activation of innate immune pathways (Zhao et al., 2019; Zhang et al., 2026). Many investigational RNA therapeutics also have failed due to insufficient delivery efficiency, inadequate target specificity, immune-mediated toxicity or poor pharmacokinetic properties (Dwivedi, 2026). These challenges are particularly evident in miRNA therapeutics and oncology-focused RNA medicines. Unlike approved siRNA drugs that generally target a single disease-associated transcript, miRNAs regulate multiple genes and pathways simultaneously, increasing the risk of off-target effects. Cancer presents additional barriers including tumour heterogeneity, inefficient penetration into solid tumours, rapid clearance and immunosuppressive tumour microenvironments (Zhang et al., 2026). Consequently, the several RNA-based oncology programs demonstrated promising preclinical efficacy but failed to achieve durable clinical benefit in patients. The clinical experience from approved RNA therapeutics indicates that successful translation requires a combination of validated molecular targets, optimized chemistry, efficient delivery platforms, durable pharmacological activity and acceptable safety profiles. Continued advances in RNA engineering and tissue-specific delivery technologies are expected to expand RNA therapeutics beyond rare genetic and metabolic diseases toward broader applications in oncology, cardiovascular diseases and neurodegenerative disorders (van Rooij and Olson, 2012; John et al., 2025) (Table 11).

**TABLE 11:** Mechanistic features associated with successful and unsuccessful RNA therapeutics.

Parameter	Approved RNA therapeutics	Failed/Unapproved candidates
Target selection	Strongly validated disease-driving genes	Often incompletely validated
Specificity	High target specificity	Broader target spectrum
Delivery	LNPs, GalNAc, optimized formulations	Inefficient tissue delivery
Stability	Extensive chemical modifications	Rapid degradation
Immune activation	Controlled	Frequently problematic
Pharmacokinetics	Durable activity	Limited exposure
Clinical efficacy	Consistent and reproducible	Variable or transient
Representative examples	Patisiran, Inclisiran, Nusinersen, Tofersen, Olezarsen, Comirnaty	MRX34, RG-101, Pegnivacogin, several oncology miRNA candidates

## 12 Future perspective and conclusion

The rapid evolution of RNA therapeutics has transformed the treatment landscape for a broad spectrum of diseases, ranging from rare genetic disorders to infectious diseases, cancer, and metabolic conditions. The clinical success of ASOs, siRNA-based therapeutics, and mRNA vaccines has validated RNA as a versatile therapeutic modality capable of targeting disease pathways that were previously considered difficult or impossible to modulate using conventional small molecules or biologics. Despite these achievements, several scientific, technological, and regulatory challenges continue to influence the broader clinical translation of RNA-based medicines.

One of the most significant barriers across all RNA therapeutic platforms remains efficient and tissue-specific delivery. Although LNPs and ligand-conjugated systems such as GalNAc have demonstrated remarkable success in delivering RNA therapeutics to the liver, extending these strategies to extrahepatic tissues remains challenging. Limited cellular uptake, endosomal entrapment, off-target accumulation, and variability in biodistribution can significantly affect therapeutic efficacy and safety.

Safety considerations also remain central to the future development of RNA therapeutics. Immune activation, unintended gene modulation, dose-dependent toxicities, and long-term safety profiles require continued evaluation, particularly for therapies intended for chronic administration. While advances in chemical modification have substantially improved RNA stability and reduced immunogenicity, further optimization is required to enhance therapeutic durability while minimizing adverse effects. In addition, large-scale manufacturing, product stability, storage requirements, and cost-effective distribution continue to present practical challenges that may influence global accessibility and widespread clinical implementation.

The future of RNA therapeutics is expected to extend well beyond currently approved modalities. Emerging technologies such as circRNAs, self-amplifying RNAs, programmable RNA editing systems, and RNA-based gene regulation platforms offer opportunities to overcome some of the limitations associated with existing approaches. circRNAs, in particular, have attracted considerable attention because of their enhanced stability and prolonged protein expression compared with conventional linear mRNA. Similarly, advances in RNA editing technologies may enable transient correction of disease-causing mutations without permanently altering the genome, potentially providing a safer alternative to DNA-based gene editing strategies.

Another promising direction involves the integration of RNA therapeutics with complementary treatment modalities. Combining RNA-based approaches with gene editing technologies, immunotherapies, or targeted molecular therapies may enhance therapeutic efficacy and address complex disease mechanisms through multiple pathways simultaneously. Furthermore, the increasing availability of genomic and transcriptomic data is expected to accelerate the development of personalized RNA medicines tailored to individual molecular profiles, thereby improving treatment precision and clinical outcomes.

Among these emerging approaches, RNA editing technologies have attracted considerable attention due to their potential to correct disease-associated genetic alterations at the RNA level without permanently modifying the genome. CRISPR/Cas-based RNA editing systems, particularly those employing RNA-targeting Cas proteins such as Cas13, enable programmable and reversible modification of RNA transcripts. These approaches offer opportunities for the treatment of genetic, neurological, and infectious diseases while potentially avoiding some of the safety concerns associated with permanent DNA editing.

Another rapidly evolving area is saRNA technology, which incorporates replication machinery derived from alphaviruses to enable intracellular amplification of RNA transcripts. Compared with conventional mRNA therapeutics, saRNA platforms can achieve prolonged and enhanced protein expression at substantially lower doses, thereby improving manufacturing efficiency and reducing production costs. Recent advances in saRNA vaccine development have further highlighted the clinical potential of this technology for infectious diseases, cancer immunotherapy, and protein replacement applications.

The increasing integration of genomic sequencing, transcriptomic profiling, and artificial intelligence-driven therapeutic design is also accelerating the emergence of personalized RNA medicines. These approaches enable the development of patient-specific therapies tailored to individual genetic mutations, molecular signatures, and disease characteristics. Personalized RNA therapeutics have already demonstrated promise in rare genetic disorders and precision oncology, where individualized treatment strategies may provide improved therapeutic outcomes compared with conventional approaches. Continued advances in RNA engineering, delivery technologies, and computational biology are expected to further expand the scope and clinical impact of these next-generation RNA-based therapies.

In conclusion, RNA therapeutics represent a transformative advancement in modern medicine, providing unprecedented opportunities to modulate gene expression, replace defective proteins, and target disease processes at the molecular level. Continued progress in RNA engineering, delivery technologies, synthetic biology, and computational approaches is expected to further expand the therapeutic scope of these modalities. Although important challenges remain, the remarkable advances achieved over the past decade strongly suggest that RNA-based medicines will play an increasingly important role in the future of precision medicine and the treatment of diverse human diseases.
